# Prevalence of Heterotrophic Methylmercury Detoxifying Bacteria across Oceanic Regions

**DOI:** 10.1021/acs.est.1c05635

**Published:** 2022-03-04

**Authors:** Isabel Sanz-Sáez, Carla Pereira-García, Andrea G. Bravo, Laura Trujillo, Martí Pla i Ferriol, Miguel Capilla, Pablo Sánchez, Rosa Carmen Rodríguez Martín-Doimeadios, Silvia G. Acinas, Olga Sánchez

**Affiliations:** † Departament de Biologia Marina i Oceanografia, Institut de Ciències del Mar, ICM-CSIC, 08003 Barcelona, Catalunya, Spain; ‡ Departament de Genètica i Microbiologia, Facultat de Biociències, Universitat Autònoma de Barcelona, 08193 Bellaterra, Spain; § Research Group in Environmental Engineering (GI2AM), Department of Chemical Engineering, University of Valencia, Av. De la Universitat S/N, 46100 Burjassot, Spain; ∥ Environmental Sciences Institute (ICAM), Department of Analytical Chemistry and Food Technology, University of Castilla-La Mancha, Avda. Carlos III s/n, 45071 Toledo, Spain

**Keywords:** mercury, methylmercury, marine bacteria, mercury-resistant bacteria, merA, merB, minimum inhibitory concentration (MIC)

## Abstract

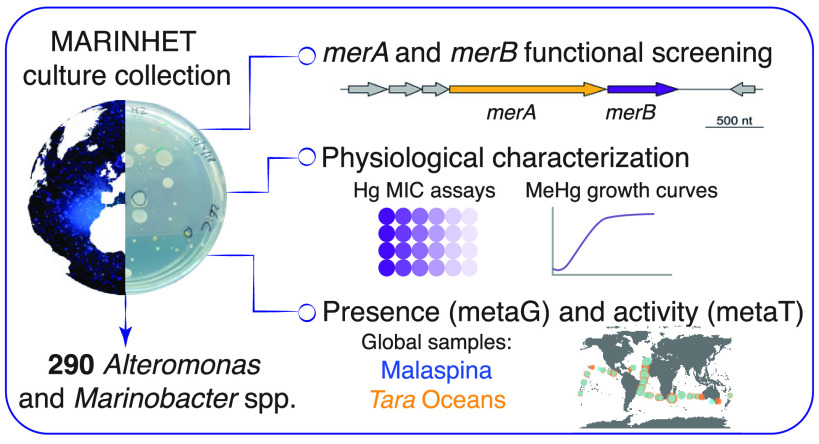

Microbial reduction of inorganic divalent mercury (Hg^2+^) and methylmercury (MeHg) demethylation is performed by the *mer* operon, specifically by *merA* and *merB* genes, respectively, but little is known about the mercury tolerance capacity of marine microorganisms and its prevalence in the ocean. Here, combining culture-dependent analyses with metagenomic and metatranscriptomic data, we show that marine bacteria that encode *mer* genes are widespread and active in the global ocean. We explored the distribution of these genes in 290 marine heterotrophic bacteria (*Alteromonas* and *Marinobacter* spp.) isolated from different oceanographic regions and depths, and assessed their tolerance to diverse concentrations of Hg^2+^ and MeHg. In particular, the *Alteromonas* sp. ISS312 strain presented the highest tolerance capacity and a degradation efficiency for MeHg of 98.2% in 24 h. Fragment recruitment analyses of *Alteromonas* sp. genomes (ISS312 strain and its associated reconstructed metagenome assembled genome MAG-0289) against microbial bathypelagic metagenomes confirm their prevalence in the deep ocean. Moreover, we retrieved 54 *merA* and 6 *merB* genes variants related to the *Alteromonas* sp. ISS312 strain from global metagenomes and metatranscriptomes from *Tara* Oceans. Our findings highlight the biological reductive MeHg degradation as a relevant pathway of the ocean Hg biogeochemical cycle.

## Abstract 2

Active mercury-resistant genes detected in marine cultured bacteria are widely distributed in the ocean, including the bathypelagic zone.

## Introduction

Mercury (Hg) is one of the most toxic, widespread, and worrisome contaminants^1,2^ and is emitted to the atmosphere by natural sources, such as volcanoes and rock weathering, but particularly by anthropogenic activities. The rising Hg levels since the industrial era, estimated as an increase of 450% in the atmosphere,^3^ makes the study of its biogeochemical cycle a major concern to the scientific community. As a consequence, the Minamata Convention, a global treaty to protect human and wildlife health and the environment from the adverse effects of mercury by, for example, reducing its atmospheric emissions, was held in 2013 and entered into force in August 2017.^4^

Emitted elemental (Hg^0^) and inorganic divalent (Hg^2+^) Hg can be deposited on land and in oceans by wet and dry depositions.^5,6^ Inorganic divalent Hg in the ocean can then be volatilized back again to the atmosphere as Hg^0^^7^ or can be methylated^8−12^ to methylmercury (MeHg), which bioaccumulates and biomagnifies in aquatic food webs.^3,13,14^ As a consequence, humans are exposed to this neurotoxicant mainly through fish and seafood consumption.^13,15,16^ Methylmercury levels in the oceans vary with depth, and usually, measures are being reported low in open ocean surface waters, maximal in intermediate layers, especially in regions of low-oxygen and near or below the thermoclines (up to 1000 m depth), and low and relatively constant in deeper waters (>1000 m depth).^13,17,18^ While Hg^2+^ methylation has been reported to occur in oxic and sub-oxic layers of the ocean water column^8,17,19−21^ mainly associated with the microbial remineralization of sinking particulate organic matter,^17,18,22^ much less is known about MeHg demethylation and Hg^2+^ reduction processes. Although MeHg demethylation and Hg^2+^ reduction processes can be photochemically mediated,^23−25^ light penetration in the ocean water column is limited to 200 m;^26^ thus, biological MeHg degradation and Hg^2+^ reduction processes likely govern in the ocean water column. Moreover, taking into account that different concentrations of MeHg can be found through the ocean water column,^20,27−29^ it would be plausible to find microorganisms with Hg detoxification capacities, especially in deep aphotic zones. However, very few studies have unveiled and demonstrated the role of microorganisms in MeHg degradation in the vast deep ocean.

Biological MeHg demethylation and Hg^2+^ reduction detoxification processes can be mediated by different biotic processes, including reductive reactions mediated by the *mer* operon,^30,31^ but also oxidative reactions^32,33^ as well as the recently described MeHg degradation performed by methanotrophic bacteria.^34^ In this article, we will focus only on the study of bacteria carrying the *mer* operon (i.e., biological reductive MeHg degradation pathways). While the operon can be composed of different sets of genes,^30,31^ the key genes are *merA* and *merB*. The first one codifies a mercuric reductase and is responsible for the transformation of Hg^2+^ to the less harmful and volatile Hg^0^.^30^ The *merB* gene encodes an organomercurial lyase enzyme that confers resistance to the organic MeHg form. It is responsible for its demethylation, releasing Hg^2+^, which is then reduced to Hg^0^ by the *merA* gene.^30^ These machineries have been found in numerous microorganisms, including aerobic and anaerobic microbial species, although demethylation appears to be predominantly accomplished by aerobic organisms.^31,33^ To date, very few studies have showed the presence of *mer* genes in oceanic waters, with the exception of some studies in the North Pacific and Arctic Oceans.^35^ In order to build this gap in knowledge, the aim of this study was to demonstrate that marine bacteria that encode *mer* genes could degrade MeHg and to explore its prevalence in the open ocean, including deep-ocean waters. For this, we took advantage of the MARINHET^36^ culture collection, which includes marine bacterial strains from a wide variety of oceanographic regions and depths. We analyzed 290 marine heterotrophic bacteria in order to: (i) detect the presence of *merA* and *merB* genes in different oceanographic regions and depths, (ii) assess the tolerance of an important fraction of strains that encode those genes to different concentrations of divalent inorganic Hg (Hg^2+^) and monomethylmercury (MeHg), (iii) describe the degradation potential for the most tolerant strains, and (iv) explore its prevalence and biogeography patterns across oceans and depths by retrieving *merA* and *merB* genes from marine microbial metagenomes (metaG) and metatranscriptomes (metaT) from the *Tara* Oceans expedition.^37,38^

Identification of Hg-resistant bacteria in contrasting aquatic ecosystems and the assessment of their tolerance to different concentrations of MeHg provide new opportunities to explore the ubiquity and prevalence of marine cultured bacteria with detoxification capacity in the open ocean (i.e., non-contaminated sites). On the other hand, this type of study sets the fundamentals for finding suitable microorganisms to be used for bioremediation strategies.

## Materials and Methods

### Primer Design for *merA* and *merB* Genes of *Alteromonas* and *Marinobacter*

Primers were designed in order to identify mercury-resistant bacteria among *Alteromonas* and *Marinobacter* strains in a culture collection because: (i) these genera are well known to encode the *mer* operon^39−43^ in their genomes; (ii) they are the most common culturable heterotrophic bacteria living in open marine waters all around the world, as they have been isolated from a wide variety of marine environments,^41,44−49^ and in the case of *Alteromonas,* it is one of the most ubiquitous cultured taxa in the ocean;^36^ and (iii) we have access to a large number of isolates thanks to the MARINHET culture collection (see the Supporting Information for more details about the culture collection). For all these reasons, these two genera were a suitable target to prove that *mer* genes can be found across different oceanographic regions and depths but also to demonstrate the MeHg-degrading capability of different strains.

Specific primer pairs were designed separately for: (i) *merA* of *Alteromonas*, (ii) *merA* of *Marinobacter*, (iii) *merA* + *merB* of *Alteromonas*, and (iv) *merA* + *merB* of *Marinobacter* based on reference sequences downloaded in 2016 from the Integrated Microbial Genomes (IMG) database of the Joint Genome Institute (JGI). See Figure S1 and Table S1 for detailed information. Noteworthy, the primers designed in this study are useful for detecting exclusively *Alteromonas* and *Marinobacter* and might not be suitable for other taxa.

### DNA Extraction and PCR Conditions

The primers previously designed were used for the screening of *merA* and *merAB* Hg-resistant genes in all *Alteromonas* and *Marinobacter* strains (*n* = 290) available at the MARINHET culture collection in 2016 (*n* = 1313). DNA of all the strains was extracted from 48 h liquid cultures grown in a Zobell broth medium (i.e., 5 g peptone, 1 g yeast extract in 750 mL of 30 kDa filtered seawater and 250 mL of Milli-Q water) using the DNeasy Blood & Tissue kit (Qiagen) following the manufacturer’s recommendations. Detailed PCR conditions are described in the Supporting Information. The PCR products were verified and quantified by agarose gel electrophoresis with a standard low-DNA mass ladder (Invitrogen). Purification and OneShot Sanger sequencing of *merA* and *merAB* genes products were performed by Genoscreen (Lille, France) with both forward and reverse primers. Geneious software v.11.0.5^50^ was used for manual cleaning and quality control of the sequences.

### Minimum Inhibitory Concentration Experiments

A total of 73 strains from the 290 isolates previously screened by PCR were subjected to minimum inhibitory concentration (MIC) assays, including all strains with positive results for *merA* and/or *merAB* presence except one that could not grow again from the cryostock. MIC assays were designed based on previous studies^43,51^ in order to assess the tolerance of the marine strains to different concentrations of inorganic Hg [as mercury(II) chloride, HgCl_2_] and organic Hg (as methylmercury chloride, CH_3_HgCl) and thus to test the activity of *merA* and *merB* genes, respectively. A stock solution of HgCl_2_ was prepared at 500 μM with autoclaved Milli-Q water. Liquid cultures of the strains growing in the Zobell broth with an optical density (O.D. at 600 nm) of 0.1 were placed in 24-well plates and inoculated with 5, 10, 20, 25, and 50 μM HgCl_2_. In specific cases, growth was observed in all HgCl_2_ concentrations and further MIC assays were done to increase the final concentrations to 50, 60, 70, 80, 90, and 100 μM. The tolerance to CH_3_HgCl was also tested for the most tolerant strains to HgCl_2_. In these case, 24-well plates were inoculated with the stock solution to reach final concentrations of 2.5, 5, 10, 15, and 20 μM. In all plates, a positive control (liquid culture of the strains not amended with CH_3_HgCl or HgCl_2_) and a negative control (broth media without bacteria in order to check for possible environmental contamination) were included in the assays. Plates were sealed with parafilm and incubated at room temperature (RT, ∼20 °C) and kept in the dark for 72 h. Visual examination and OD measurements at 600 nm were done in a 24 h period using an automatic plate reader (Infinite M200, Tecan), and data were collected using the Magellan Data Analysis Software (Tecan Diagnostics).

### Growth Curves

Growth curves were performed to characterize the growth rates of the most tolerant strain (ISS312) to different concentrations of CH_3_HgCl. We prepared 200 mL of liquid cultures in the Zobell broth supplemented with CH_3_HgCl at final concentrations of 0 (positive control), 1, 2.5, and 5 μM in triplicates. The initial OD at 600 nm of the cultures was 0.05 in order to assure enough concentration of cells for growth. Samples for OD measurements and for bacterial cell counts were taken approximately every 2 h. OD was measured at 600 nm with a spectrophotometer (Varian Cary 100 UV–Vis), and cells were stained with 4′,6-diamidino-2-phenylindole and counted with an automated microscope Zeiss Axio Imager Z2M^52,53^ using the automated image analysis software ACME Tool (www.technobiology.ch). Predicted growth curves based on OD observations and kinetic values, including growth rates (μ_max_), carrying capacity (k), and lag phase time, were calculated with the R package Growthcurver v.0.3.0^54^ and GrowthRates v.4.3 software.^55^ For graphical representation, replicates of the different growth curve experiments at several CH_3_HgCl concentrations were averaged. Hence, mean OD and standard deviation were calculated for each time point of the curves.

From the 1 and 5 μM growth curves, 2 mL samples were also taken for characterizing CH_3_HgCl degradation rates at time 0, 6, 12, 24, and 48 h. Besides, in order to check the possibility that CH_3_HgCl was being abiotically removed, we also measured the CH_3_HgCl concentrations from samples taken from multiwell plate experiments, including different controls (more details in the Supporting Information). Descriptions of Hg species concentration measurements are also provided in the Supporting Information.

### Phylogenetic Analyses

In order to show that MeHg degradation capacity is strain specific, a phylogeny of the isolates screened by PCR for *merA* and *merAB* genes was inferred from their partial 16S rRNA sequences in order to detect a possible clustering between all the positive strains. The closest sequence to each isolate 16S rRNA gene was obtained by BLASTn^56^ against the SILVA v.132 database. Alignment of the isolates and reference sequences was performed with MUSCLE from the Geneious software v.11.0.5.^50^ The alignment was trimmed to the common 16S rRNA gene fragment covered by both sets of sequences. Phylogeny was constructed using maximum-likelihood inference with RAXML-NG 0.9.0,^57^ the GTR evolutionary model with optimization in the among-site rate heterogeneity model and the proportion of invariant sites (GTR + G + I), and 100 bootstrap replicates. In the same way, a phylogenetic tree was constructed with the partial 16S rRNA sequences of the positive isolates only. In this tree, the closest match in the SILVA v.132 database was also included. Presence of *merA* and *merAB* genes, origin of the strains, plus their tolerance to HgCl_2_ and MeHg were added with Interactive Tree of Life (ITOL).^58^ Supplementary phylogenetic trees were also constructed using amino acid sequences of *merA* and *merB* genes (see the Supporting Information for details).

### Fragment Recruitment Analyses of the Genome of ISS312 strain and MAG-0289 in Bathypelagic Metagenomes

The abundance of ISS312 strain across the global bathypelagic ocean was assessed thanks to the sequencing of its complete genome (Supporting Information) that it was taxonomically assigned to*Alteromonas mediterranea*. In addition, thanks to a previous study,^59^ we could reconstruct a metagenome assembled genome (MAG-0289) also affiliated to the *Alteromonas mediterranea* and encoding *merA* and *merB* genes. Fragment recruitment analyses (FRA) of the ISS312 strain and the MAG-0289 was performed by mapping the metagenomic reads of 58 bathypelagic microbial metaG from 32 stations^59,60^ from the Malaspina expedition, including free-living (FL) (0.2–0.8 μm) and particle-attached (PA) (0.8–20 μm) microbial communities. Analyses were done with BLASTn v2.7.1+.^56^ Details of FRA are explained in the Supporting Information.

### Detection of *merA* and *merB* Genes in Global Metagenomes and Metatranscriptomes from *Tara* Oceans Expedition

BLASTp analyses were performed using a conservative *e-value* (>1E-100) with the *merA* and *merB* sequences of the ISS312 genome against global prokaryotic metaG and metaT available in the Ocean Microbial Reference Gene catalog V2 (OMRGC.v2)^38^ from the *Tara* Oceans expedition covering surface, deep chlorophyll maximum (DCM), and mesopelagic layers across oceanographic regions. The generation and annotation of OMRGC.v2, the taxonomic profiling of metagenomic and metatranscriptomic composition, and the normalization of metagenomic and metatranscriptomic profiles used for the extraction of the abundance of the *merA* and *merB* genes homologous were thoroughly explained in Salazar et al.^38^ Analyses could be performed thanks to the Ocean Gene Atlas Web resource,^37^ and abundance results from metaG and metaT were normalized based on the percentage of mapped reads.

### Statistical Analyses

ANOVA tests from the stats package of the R statistical software^61^ were performed in order to observe if the presence of *merA* and/or *merAB* genes in the studied strains was linked to specific oceanographic locations. Further, non-parametric Kruskal–Wallis test, from the stats package of the R statistical software,^61^ was performed followed by the post hoc pairwise Wilcox test to see the differences between FRA results in different oceanographic regions and between FL and PA bacterial communities. To assess significance, all the statistical analyses were set to an alpha value of 0.05.

### Nucleotide Accession Numbers

Mercury detoxification genes (*merA* and *merAB*) detected in this study through PCR were deposited in GenBank under accession numbers MW273028–MW273125. The *A. mediterranea* ISS312 genome was deposited in ENA under study accession number PRJEB46669.

## Results and Discussion

### Presence of *merA* and *merB* Genes among *Alteromonas* and *Marinobacter* Trains

Among the 290 strains of the MARINHET bacterial culture collection,^36^ 244 were taxonomically classified as *Alteromonas* sp. and 46 as *Marinobacter* sp. (Table S2). These strains were isolated from different depths, including the surface, the deep chlorophyll maximum (DCM), and the bathypelagic zone. The strains here studied for *merAB* genes were isolated from different oceanic regions such as the North Western Mediterranean Sea (89), South (101) and North Atlantic (42), Indian (44), Arctic (7) and Southern (7) Oceans and included isolates from photic (160) and aphotic (130) layers (Table 1, Figure S2, and Tables S3, S4).

**Table 1:** Summary of the PCR Screening Results for *merA* and *merAB* in *Alteromonas* and *Marinobacter* Strains^a^

				positives PCR for		
genus	no. of tested strains	layer	ocean	*merA*	*merAB*	total strains with *merA* and/or *merAB*
*Alteromonas*	127	photic	Southern Ocean	1	0	33 (13.5%)
			Indian Ocean	0	1	
			NW Mediterranean	2	0	
			North Atlantic Ocean	5	0	
			South Atlantic Ocean	4	0	
	117	aphotic	South Atlantic Ocean	18	3	
*Marinobacter*	33	photic	Southern Ocean	4	4	41 (89.1%)
			North Atlantic Ocean	1	0	
			South Atlantic Ocean	26	16	
	13	aphotic	NW Mediterranean	9	2	
			North Atlantic Ocean	1	0	
			South Atlantic Ocean	1	0	

a Photic includes surface and DCM isolates, while aphotic includes bathypelagic isolates. NW Mediterranean: North Western Mediterranean.

The functional screening of the *merA* and *merB* genes from the 244 *Alteromonas* and 46 *Marinobacter* strains revealed that 13.5% (32 out of 244) and 89.1% (41 out of 46) of the strains presented only *merA*, while only 1.6% (4 out of 244) and 47.8% (22 out of 46) presented both *merA* and *merB* genes (*merAB*) (Table 1). No significant differences were found between depths or between oceans (ANOVA, *P*-value > 0.05), but in general, we found a higher proportion of positive strains coming from waters of the Southern Ocean (71% despite the lower number of strains tested) and the South Atlantic Ocean (48%), followed by those retrieved from the North Atlantic Ocean (17%) and the North Western Mediterranean Sea (13%).

### High Variability of Mercury Tolerance within Marine Bacteria

**Figure 1:**
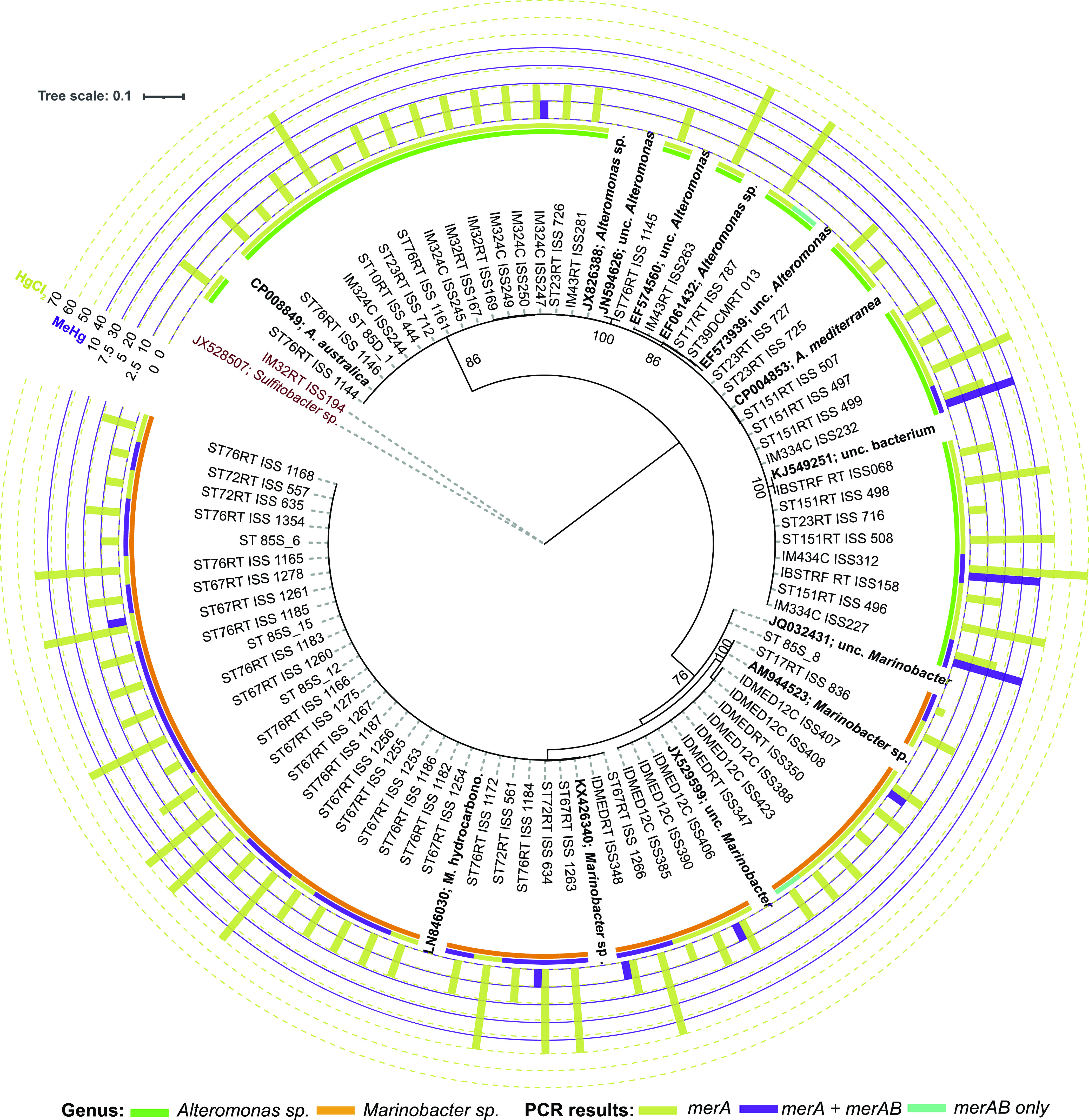
Phylogeny of the 16 S rRNA gene of *Alteromonas* and *Marinobacter* positive strains for *merA* and/or *merAB* genes screening. First inner colored strip indicates genus of the strain. Second colored strip indicates the presence or absence of genes based on PCR results. Bars indicate results from the MIC experiments: yellow-green, HgCl_2_; purple, MeHg. Tolerance values are in μM. JX52807, *Sulfitobacter,* and IM32RT_ISS194 are outgroups of the tree. The numbers in the nodes represent bootstrap percentages >75%. Names in bold indicate reference sequences: *A. australica*, *Alteromonas australica*; *A. mediterranea*, *Alteromonas mediterranea*; unc, uncultured; *M. hydrocarbono*, *Marinobacter hydrocarbonoclasticus*.

It is unknown whether mercury tolerance is a conservative trait within marine bacterial strains of the same genera. To prove so, MIC experiments were done in all *Alteromonas* and *Marinobacter* isolates (73 strains) presenting *merA* and/or *merAB* detected by PCR, except one isolate that was not able to grow again from the cryostock. First, we tested the tolerance for inorganic mercury (HgCl_2_), and we observed that 32 *Alteromonas* and 41 *Marinobacter* strains displayed different levels of tolerance. MIC values ranged generally from 5 to 50 μM. Around 50% of the *Alteromonas* and *Marinobacter* strains tested presented a MIC of 20 μM, and one of the isolates stood out as it presented a tolerance to HgCl_2_ up to 70 μM (Table S5). Besides, we tested the tolerance to MeHg amended in the form of CH_3_HgCl for those strains already presenting a tolerance to inorganic mercury above 20 μM and encoding the *merB* gene. For *Alteromonas*, three strains taxonomically classified as *A. mediterranea* presented a high tolerance to CH_3_HgCl, growing at concentrations up to 10 μM (Figure 1), but all the tested *Marinobacter* sp. strains did not show a substantial growth above 2.5 μM of CH_3_HgCl (Figure 1).

If we take a look into the phylogenetic tree constructed with the 16S rRNA sequences (Figure 1) of the strains presenting *merA* and/or *merAB* genes, different tolerances were found within the same phylogenetic cluster. For example, within the cluster of *A. mediterranea,* some strains presented a MIC of 20 μM, while the isolate that presented the highest tolerance (70 μM, ISS312) also belonged to the same *Alteromonas* species (Figure 1). The same pattern occurred among *Marinobacter* isolates, where members of the *Marinobacter hydrocarbonoclasticus* cluster presented MIC values ranging from 10 to 50 μM (Table S5 and Figure 1). The same pattern can be observed in the *merA* and *merAB* amino acid phylogenies (Figures S3 and S4).

The MIC value heterogeneity within strains belonging to the same phylogenetic cluster suggested that the level of Hg resistance was strain specific, and we probably retrieved different ecotypes within the same species with contrasting tolerances to Hg. Despite these differences between strains, the tolerances found for were similar to those found in other studies where *Alteromonas*(43,62−64) and *Marinobacter*(65) genera were also isolated from different marine ecosystems such as hydrothermal vents, estuaries, or contaminated sediments. However, to the best of our knowledge, this is the first study that addresses the tolerance of *Marinobacter* spp. and *A. mediterranea* isolated from the ocean to MeHg. Hence, we found out that a strain affiliated to *A. mediterranea* (ISS312) presented a MIC to HgCl_2_ higher than other strains already published, up to 70 μM, and we also determined that it was able to grow in the presence of MeHg, presenting a MIC up to 10 μM, representing a good candidate for future bioremediation studies in highly contaminated areas with both organic and inorganic mercuric compounds.

### Description of the Highly Tolerant *Alteromonas* sp. Strain ISS312

**Figure 2:**
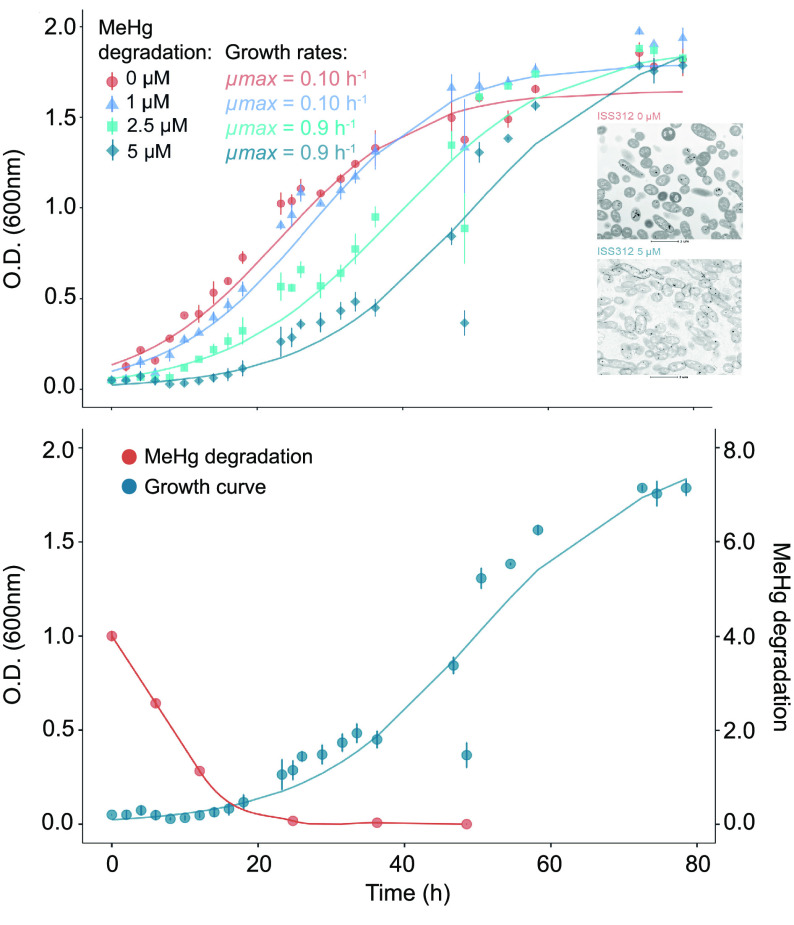
Growth effect of MeHg in ISS312 strain. (A) Growth kinetics of the *A. mediterranea* ISS312 strain in a Zobell broth containing MeHg [control (0 μM), 1, 2.5, and 5 μM]. μ_max_ indicates the maximum growth rate for each MeHg concentration. TEM images of the strain growing at 0 μM and 5 μM are shown in the right side of the plot. Details for preparation and observation of samples for TEM are explained in the Supporting Information. (B) MeHg removal by strain ISS312 during the growth curve experiment at 5 μM. Mean and standard deviation from three replicates samples are shown in both graphs.

Strain ISS312, isolated from South Atlantic bathypelagic waters at 4000 m and classified as *A. mediterranea*, displayed the highest tolerance to both HgCl_2_ (70 μM) and MeHg (10 μM). The growth rates of this strain at different concentrations of MeHg were assessed and included: a control without MeHg (0 μM) and with 1, 2.5, and 5 μM MeHg. Growth curves at 0 and 1 μM were very similar, as well as between 2.5 and 5 μM (Figure 2A). We observed that the major difference between growth curves was the length of the lag phase, where bacteria adapt themselves to the growth conditions. This phenomenon seems to be a common trait for Hg-resistant strains in the presence of toxic compounds, as this behavior has been repeatedly observed in different species of *Pseudomonas* sp., *Alcaligenes* sp., or *Bacillus* sp.^66−68^ However, once the cultures started to grow, their growth rates (μ_max_) were very similar independently of their initial MeHg concentrations, ranging from 0.10 h^–1^ in the control to 0.09 h^–1^ at 5 μM. A stationary phase was reached in all concentrations at 80 h, even though at this time, cultures at higher concentrations of MeHg seemed to be only entering the plateau (Figure 2A). In addition, their carrying capacity (*k*), that is, the maximum population size of a species, was between 1.6 and 1.9 based on OD measures, revealing very similar values between tested concentrations, an observation also recurrently reported.^67,69^ Transmission electron microscopy (TEM) observations of the ISS312 cultures growing at 0 and 5 μM of MeHg also showed similar morphology and ultrastructure of the cells (Figure 2A).

MeHg concentrations were measured in the isolate exposed to 5 μM at different incubation times. MeHg concentrations were reduced by 36% (2.6 μM) and 72% (1.1 μM) (Figure 2B) during the lag phase at 6 h and 12 h, respectively. After 24 h, when almost all MeHg was removed (98.2%), this microorganism began the exponential growth phase. After 48 h, MeHg could not be detected (Figure 2B and Table S6). It is important to highlight that we detected a certain level of abiotic MeHg degradation (up to 25%) in the medium exposed without bacteria and in the killed control (Table S7).

### Global Distribution of the ISS312 Strain and MAG-0289 in the Bathypelagic Ocean

**Figure 3:**
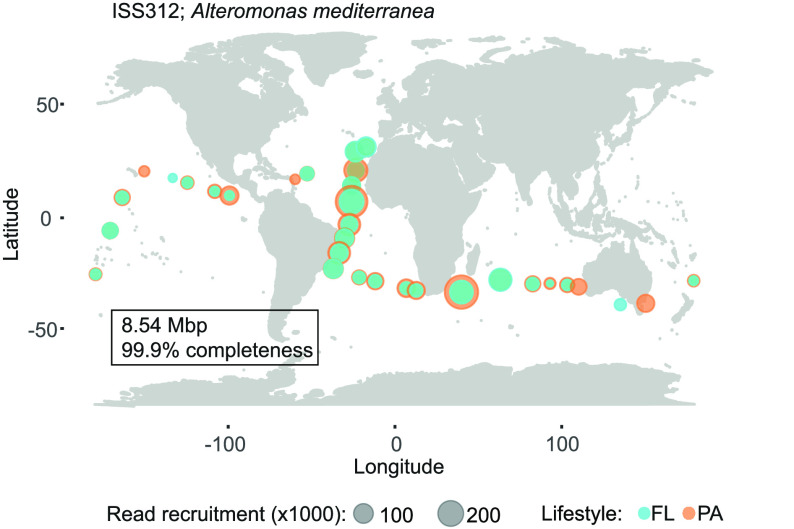
World map showing the distribution of the *A. mediterranea* strain ISS312. Size of the dots indicate number of reads (×1000) and color indicates if the reads were recruited in the FL (0.2–0.8 μm) or in the PA (0.8–20 μm) microbial communities of the bathypelagic samples.

The biogeographic and size fraction distribution of the ISS312 genome was assessed in all available bathypelagic metaG of the Malaspina expedition^59^ because strain ISS312 was originally retrieved from bathypelagic waters of the South Atlantic Ocean. We found that this strain belonging to the *A. mediterranea* species was distributed across all the temperate bathypelagic waters, including the Atlantic, the Pacific, and the Indian Oceans (Figure 3). Its abundance, according to the data from the FRA, varied across ocean basins, and we found significant differences between the Pacific and the Brazil basins (*P*-value = 0.019), and between the Pacific and the Canary basins (*P*-value: 0.011), suggesting a higher abundance of this bacterium in the Atlantic Ocean. Despite finding these differences between oceans, we did not find significant differences between plankton size fractions, indicating that the isolate could be present in both the FL (0.2–0.8 μm) and the PA (0.8–20 μm) microbial communities (Figure 3). Moreover, by using bathypelagic metaG, one MAG was reconstructed, the MAG-0289, which genome-aligned at 99% with the *Alteromonas* sp. ISS312 strain genome and showed a 99.34% average nucleotide identity. The FRA of the MAG-0289 and the ISS312 strain against the bathypelagic metaG displayed identical biogeographical patterns (Figure S5), indicating that the MAG-0289 is a good representative of the ISS312 strain.

Our results confirm the prevalence and wide distribution of the *A. mediterranea* species carrying Hg-resistant genes across the global bathypelagic ocean but also shows its occurrence in both plankton microbial size fractions analysed. To the best of our knowledge, the detection and characterization of microorganisms encoding for*mer* genes have not been previously reported from non-contaminated deep open ocean waters at a large scale.

### Recovery of *merA* and *merB* Genes from Related Lineages of ISS312 from Marine Microbial Metagenomes and Metatranscriptomes

**Figure 4:**
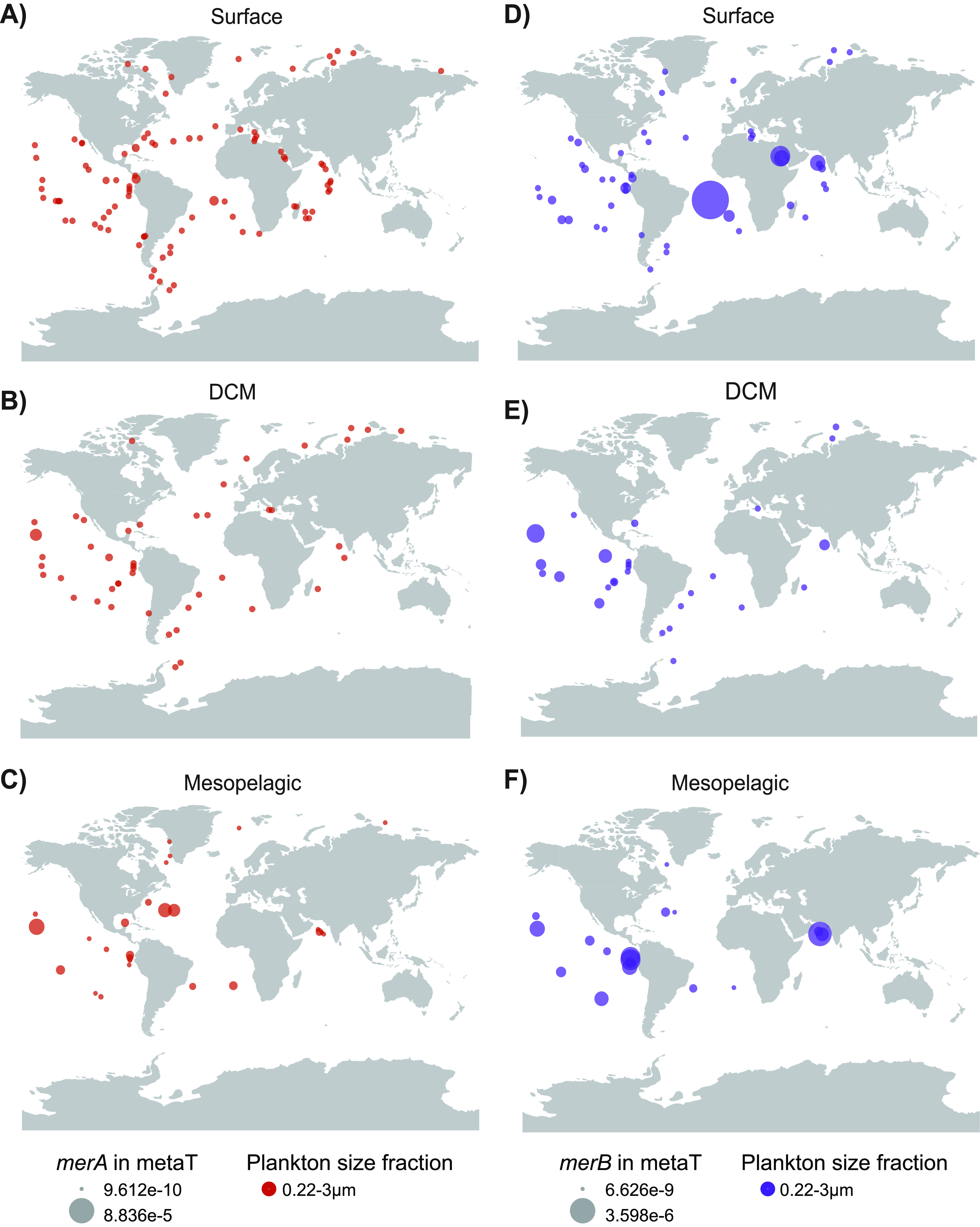
Biogeography of the *merA* and *merB* transcripts across oceanic regions and depths from microbial metaT from the *Tara* Oceans expedition. (A–C) Abundance of the *merA* transcripts found on the surface, DCM, and mesopelagic samples. (D–F) Abundance of *merB* transcripts found on the surface, DCM, and mesopelagic samples.

A remaining question is whether *merA* and *merB* genes are active under natural ambient Hg concentrations. For answering this question, we have analyzed the available metaT of the Ocean Microbial Reference Gene Catalog V2 (OMRGC.v2)^38^ from the *Tara* Oceans expedition through the Ocean Gene Atlas resource^37^ covering surface, DCM, and mesopelagic layers across oceanographic regions. BLASTp search using the *merA* and *merB* genes from the ISS312 strain as input allowed us to extract 54 *merA* and 6 *merB* genes (*e-value* 1E-100) that ranged from 64 to 99.3% identities in their amino acid sequences and belonged to gammaproteobacterial lineages mainly from the *Alteromonadales* order and *Halomonadaceae* family (Table S8). These *merA* and *merB* transcripts were prevalent across oceans and depths (see Figure 4, Figures S6 and S7). We found that *merB* genes were more transcribed (Figure 4) in the mesopelagic zone of the North Indian and Eastern South Pacific Oceans, which are well known regions of oxygen minimum zone areas, and also in some stations from surface South Atlantic waters. It is known that MeHg production presents its maximum concentrations usually near the thermocline and in regions with low oxygen concentrations.^20,70^ Perhaps bacteria present in those regions encode and transcribe the Hg-resistant genes in order to cope with those more elevated MeHg concentrations.

### Outlook

A combination of culture-dependent analyses with metagenomic and metatranscriptomic analyses from the global photic and aphotic oceans unveiled that biological reductive MeHg degradation capacities were widely distributed and active in the open ocean, especially in the mesopelagic zone. In particular, this study has uncovered that heterotrophic bacteria containing *mer* genes are present in the open ocean from different oceanographic regions and depths but also plankton size fractions. Also, this study reveals that strains closely related phylogenetically presented contrasting levels of tolerance to Hg, indicating that this biological capacity is strain specific. In particular, we show that the ISS312 strain isolated from bathypelagic waters of the South Atlantic Ocean presented a strong and fast capacity to degrade MeHg. Moreover, the ISS312 strain genome and related lineages harboring *merA* and *merB* genes are present and transcribed globally in marine samples across oceans and depths, including polar regions. This outcome has important implications in the biogeochemical cycle of Hg as it provides new understanding on the main players driving MeHg levels in the ocean and can ultimately help to improve current frameworks for marine food webs and human exposure to MeHg.

## Supporting Information Available

Supplementary details for primers designd and Supplementary results for PCR screening, MIC assays, MeHg degradation assays, homologous *merA* and *merB* genes detected in metagenomes and metatranscriptomes of *Tara* Oceans gene catalog (XLSX)
Detailed description of some extra analyses and methodologies used in the study, first exploration in the MARINHET collection, description of primers used, extra phylogenetic analyses with 16S rRNA genes and extra phylogenetic trees with *merA* and *merB* genes, results from metaG and metaT analyses (PDF)

## References

[ref1] Miller M. W.; Clarkson T. W. Mercury, Mercurial, and Mercaptans; Charles C Thomas: Springfield, ILL, 1973.

[ref2] Clarkson T. W. Mercury: Major Issues in Environmental Health. Environ. Health Perspect. 1993, 100, 31–38. 10.1289/ehp.9310031. 8354179 PMC1519577

[ref3] UN Environment Global Mercury Assessment 2018; UN Environment Programme, Chemicals and Health Branch: Geneva, Switzerland, 2019.

[ref4] Eriksen H. H.; Perrez F. X. The Minamata Convention: A Comprehensive Response to a Global Problem. Rev. Eur. Comp. Int. Environ. Law 2014, 23, 195–210. 10.1111/reel.12079.

[ref5] Saiz-Lopez A.; Sitkiewicz S. P.; Roca-Sanjuán D.; Oliva-Enrich J. M.; Dávalos J. Z.; Notario R.; Jiskra M.; Xu Y.; Wang F.; Thackray C. P.; Sunderland E. M.; Jacob D. J.; Travnikov O.; Cuevas C. A.; Acuña A. U.; Rivero D.; Plane J. M. C.; Kinnison D. E.; Sonke J. E. Photoreduction of Gaseous Oxidized Mercury Changes Global Atmospheric Mercury Speciation, Transport and Deposition. Nat. Commun. 2018, 9, 4796 10.1038/s41467-018-07075-3. 30442890 PMC6237998

[ref6] Enrico M.; Roux G. L.; Marusczak N.; Heimbürger L.-E.; Claustres A.; Fu X.; Sun R.; Sonke J. E. Atmospheric Mercury Transfer to Peat Bogs Dominated by Gaseous Elemental Mercury Dry Deposition. Environ. Sci. Technol. 2016, 50, 2405–2412. 10.1021/acs.est.5b06058. 26849121

[ref7] Mason R. P.; Sheu G.-R. Role of the Ocean in the Global Mercury Cycle. Global Biogeochem. Cycles 2002, 16, 40 10.1029/2001GB001440.

[ref8] Lehnherr I.; St Louis V. L.; Hintelmann H.; Kirk J. L. Methylation of Inorganic Mercury in Polar Marine Waters. Nat. Geosci. 2011, 4, 298–302. 10.1038/ngeo1134.

[ref9] Munson K. M.; Lamborg C. H.; Boiteau R. M.; Saito M. A. Dynamic Mercury Methylation and Demethylation in Oligotrophic Marine Water. Biogeosciences 2018, 15, 6451–6460. 10.5194/bg-15-6451-2018.

[ref10] Monperrus M.; Tessier E.; Amouroux D.; Leynaert A.; Huonnic P.; Donard O. F. X. Mercury Methylation, Demethylation and Reduction Rates in Coastal and Marine Surface Waters of the Mediterranean Sea. Mar. Chem. 2007, 107, 49–63. 10.1016/j.marchem.2007.01.018.

[ref11] Podar M.; Gilmour C. C.; Brandt C. C.; Soren A.; Brown S. D.; Crable B. R.; Palumbo A. V.; Somenahally A. C.; Elias D. A. Global Prevalence and Distribution of Genes and Microorganisms Involved in Mercury Methylation. Sci. Adv. 2015, 1, e1500675 10.1126/sciadv.1500675. 26601305 PMC4646819

[ref12] Gionfriddo C. M.; Tate M. T.; Wick R. R.; Schultz M. B.; Zemla A.; Thelen M. P.; Schofield R.; Krabbenhoft D. P.; Holt K. E.; Moreau J. W. Microbial Mercury Methylation in Antarctic Sea Ice. Nat. Microbiol. 2016, 1, 16127 10.1038/nmicrobiol.2016.127. 27670112

[ref13] Mason R. P.; Choi A. L.; Fitzgerald W. F.; Hammerschmidt C. R.; Lamborg C. H.; Soerensen A. L.; Sunderland E. M. Mercury Biogeochemical Cycling in the Ocean and Policy Implications. Environ. Res. 2012, 119, 101–117. 10.1016/j.envres.2012.03.013. 22559948 PMC3427470

[ref14] Harding G.; Dalziel J.; Vass P. Bioaccumulation of Methylmercury within the Marine Food Web of the Outer Bay of Fundy, Gulf of Maine. PLoS One 2018, 13, e0197220 10.1371/journal.pone.0197220. 30011281 PMC6047777

[ref15] Mergler D.; Anderson H. A.; Chan L. H. M.; Mahaffey K. R.; Murray M.; Sakamoto M.; Stern A. H. Methylmercury Exposure and Health Effects in Humans: A Worldwide Concern. Ambio 2007, 36, 3–11. 10.1579/0044-7447(2007)36[3:MEAHEI]2.0.CO;2. 17408186

[ref16] Karagas M. R.; Choi A. L.; Oken E.; Horvat M.; Schoeny R.; Kamai E.; Cowell W.; Grandjean P.; Korrick S. Evidence on the Human Health Effects of Low-Level Methylmercury Exposure. Environ. Health Perspect. 2012, 120, 799–806. 10.1289/ehp.1104494. 22275730 PMC3385440

[ref17] Cossa D.; Averty B.; Pirrone N. The Origin of Methylmercury in Open Mediterranean Waters. Limnol. Oceanogr. 2009, 54, 837–844. 10.4319/lo.2009.54.3.0837.

[ref18] Sunderland E. M.; Krabbenhoft D. P.; Moreau J. W.; Strode S. A.; Landing W. M. Mercury Sources, Distribution, and Bioavailability in the North Pacific Ocean: Insights from Data and Models. Global Biogeochem. Cycles 2009, 23, a 10.1029/2008GB003425.

[ref19] Blum J. D.; Popp B. N.; Drazen J. C.; Anela Choy C.; Johnson M. W. Methylmercury Production below the Mixed Layer in the North Pacific Ocean. Nat. Geosci. 2013, 6, 879–884. 10.1038/ngeo1918.

[ref20] Hammerschmidt C. R.; Bowman K. L. Vertical Methylmercury Distribution in the Subtropical North Pacific Ocean. Mar. Chem. 2012, 132–133, 77–82. 10.1016/j.marchem.2012.02.005.

[ref21] Malcolm E. G.; Schaefer J. K.; Ekstrom E. B.; Tuit C. B.; Jayakumar A.; Park H.; Ward B. B.; Morel F. M. M. Mercury Methylation in Oxygen Deficient Zones of the Oceans: No Evidence for the Predominance of Anaerobes. Mar. Chem. 2010, 122, 11–19. 10.1016/J.MARCHEM.2010.08.004.

[ref22] Lamborg C. H.; Hammerschmidt C. R.; Bowman K. L. An Examination of the Role of Particles in Oceanic Mercury Cycling. Philos. Trans. R. Soc., A 2016, 374, 20150297 10.1098/rsta.2015.0297. PMC5069532 29035262

[ref23] Zhang T.; Hsu-Kim H. Photolytic Degradation of Methylmercury Enhanced by Binding to Natural Organic Ligands Tong. Nat. Geosci. 2010, 3, 473–476. 10.1038/ngeo892. 20634995 PMC2902198

[ref24] Seller P.; Kelly C. A.; Rudd J. W. M.; MacHutchon A. R. Photodegradation of Methylmercury in Lakes. Nature 1996, 380, 694–697. 10.1038/380694a0.

[ref25] Costa M.; Liss P. S. Photoreduction of Mercury in Sea Water and Its Possible Implications for Hg0 Air-Sea Fluxes. Mar. Chem. 1999, 68, 87–95. 10.1016/S0304-4203(99)00067-5.

[ref26] Stal L. J.; Cretoiu M. S. What Is so Special about Marine Microorganisms? Introduction to the Marine Microbiome - from Diversity to Biotechnological Applications. In The Marine Microbiome; Stal L. J., Cretoiu M. S., Eds.; Springer: Switzerland, 2016; pp 3–20.

[ref27] Wang F.; Macdonald R. W.; Armstrong D. A.; Stern G. A. Total and Methylated Mercury in the Beaufort Sea: The Role of Local and Recent Organic Remineralization. Environ. Sci. Technol. 2012, 46, 11821–11828. 10.1021/es302882d. 23025753

[ref28] Heimbürger L.-E.; Cossa D.; Marty J.-C.; Migon C.; Averty B.; Dufour A.; Ras J. Methyl Mercury Distributions in Relation to the Presence of Nano- and Picophytoplankton in an Oceanic Water Column (Ligurian Sea, North-Western Mediterranean). Geochim. Cosmochim. Acta 2010, 74, 5549–5559. 10.1016/J.GCA.2010.06.036.

[ref29] Gworek B.; Bemowska-Kałabun O.; Kijeńska M.; Wrzosek-Jakubowska J. Mercury in Marine and Oceanic Waters—a Review. Water, Air, Soil Pollut. 2016, 227, 371 10.1007/s11270-016-3060-3. 27656005 PMC5013138

[ref30] Barkay T.; Miller S. M.; Summers A. O. Bacterial Mercury Resistance from Atoms to Ecosystems. FEMS Microbiol. Rev. 2003, 27, 355–384. 10.1016/S0168-6445(03)00046-9. 12829275

[ref31] Boyd E. S.; Barkay T. The Mercury Resistance Operon: From an Origin in a Geothermal Environment to an Efficient Detoxification Machine. Front. Microbiol. 2012, 3, 349 10.3389/fmicb.2012.00349. 23087676 PMC3466566

[ref32] Oremland R. S.; Miller L. G.; Dowdle P.; Connell T.; Barkay T. Methylmercury Oxidative Degradation Potentials in Contaminated and Pristine Sediments of the Carson River, Nevada. Appl. Environ. Microbiol. 1995, 61, 2745–2753. 10.1128/AEM.61.7.2745-2753.1995. 16535081 PMC1388499

[ref33] Oremland R. S.; Culbertson C. W.; Winfrey M. R. Methylmercury Decomposition in Sediments and Bacterial Cultures: Involvement of Methanogens and Sulfate Reducers in Oxidative Demethylation. Appl. Environ. Microbiol. 1991, 57, 130–137. 10.1128/AEM.57.1.130-137.1991. 16348388 PMC182673

[ref34] Barkay T.; Gu B. Demethylation–The Other Side of the Mercury Methylation Coin: A Critical Review. ACS Environ. Au 2021, 10.1021/acsenvironau.1c00022. PMC10114901 37101582

[ref35] Bowman K. L.; Collins R. E.; Agather A. M.; Lamborg C. H.; Hammerschmidt C. R.; Kaul D.; Dupont C. L.; Christensen G. A.; Elias D. A. Distribution of Mercury-cycling Genes in the Arctic and Equatorial Pacific Oceans and Their Relationship to Mercury Speciation. Limnol. Oceanogr. 2019, 65, S310–S320. 10.1002/lno.11310.

[ref36] Sanz-Sáez I.; Salazar G.; Sánchez P.; Lara E.; Royo-Llonch M.; Sà E. L.; Lucena T.; Pujalte M. J.; Vaqué D.; Duarte C. M.; Gasol J. M.; Pedrós-Alió C.; Sánchez O.; Acinas S. G. Diversity and Distribution of Marine Heterotrophic Bacteria from a Large Culture Collection. BMC Microbiol. 2020, 20, 207 10.1186/s12866-020-01884-7. 32660423 PMC7359222

[ref37] Villar E.; Vannier T.; Vernette C.; Lescot M.; Cuenca M.; Alexandre A.; Bachelerie P.; Rosnet T.; Pelletier E.; Sunagawa S.; Hingamp P. The Ocean Gene Atlas: Exploring the Biogeography of Plankton Genes Online. Nucleic Acids Res. 2018, 46, W289–W295. 10.1093/nar/gky376. 29788376 PMC6030836

[ref38] Salazar G.; Paoli L.; Alberti A.; Huerta-Cepas J.; Ruscheweyh H.-J.; Cuenca M.; Field C. M.; Coelho L. P.; Cruaud C.; Engelen S.; Gregory A. C.; Labadie K.; Marec C.; Pelletier E.; Royo-Llonch M.; Roux S.; Sánchez P.; Uehara H.; Zayed A. A.; Zeller G.; Carmichael M.; Dimier C.; Ferland J.; Kandels S.; Picheral M.; Pisarev S.; Poulain J.; Acinas S. G.; Babin M.; Bork P.; Bowler C.; de Vargas C.; Guidi L.; Hingamp P.; Iudicone D.; Karp-Boss L.; Karsenti E.; Ogata H.; Pesant S.; Speich S.; Sullivan M. B.; Wincker P.; Sunagawa S.; Acinas S. G.; Babin M.; Bork P.; Boss E.; Bowler C.; Cochrane G.; de Vargas C.; Follows M.; Gorsky G.; Grimsley N.; Guidi L.; Hingamp P. Gene Expression Changes and Community Turnover Differentially Shape the Global Ocean Metatranscriptome. Cell 2019, 179, 1068–1083.e21. 10.1016/j.cell.2019.10.014. 31730850 PMC6912165

[ref39] Singer E.; Webb E. A.; Nelson W. C.; Heidelberg J. F.; Ivanova N.; Pati A.; Edwards K. J. Genomic Potential of Marinobacter Aquaeolei, a Biogeochemical “Opportunitroph”. Appl. Environ. Microbiol. 2011, 77, 2763–2771. 10.1128/AEM.01866-10. 21335390 PMC3126349

[ref40] López-Pérez M.; Gonzaga A.; Martin-Cuadrado A.-B.; Onyshchenko O.; Ghavidel A.; Ghai R.; Rodriguez-Valera F. Genomes of Surface Isolates of Alteromonas Macleodii: The Life of a Widespread Marine Opportunistic Copiotroph. Sci. Rep. 2012, 2, 696 10.1038/srep00696. 23019517 PMC3458243

[ref41] Handley K. M.; Lloyd J. R. Biogeochemical Implications of the Ubiquitous Colonization of Marine Habitats and Redox Gradients by Marinobacter Species. Front. Microbiol. 2013, 4, 136 10.3389/fmicb.2013.00136. 23734151 PMC3660661

[ref42] Fontanez K. M.; Eppley J. M.; Samo T. J.; Karl D. M.; DeLong E. F. Microbial Community Structure and Function on Sinking Particles in the North Pacific Subtropical Gyre. Front. Microbiol. 2015, 6, 469 10.3389/fmicb.2015.00469. 26042105 PMC4436931

[ref43] Ivars-Martinez E.; Martin-Cuadrado A.-B.; D’Auria G.; Mira A.; Ferriera S.; Johnson J.; Friedman R.; Rodriguez-Valera F. Comparative Genomics of Two Ecotypes of the Marine Planktonic Copiotroph Alteromonas Macleodii Suggests Alternative Lifestyles Associated with Different Kinds of Particulate Organic Matter. ISME J. 2008, 2, 1194–1212. 10.1038/ismej.2008.74. 18670397

[ref44] Baumann L.; Baumann P.; Mandel M.; Allen R. D. Taxonomy of Aerobic Marine Eubacteria. J. Bacteriol. 1972, 110, 402–429. 10.1128/jb.110.1.402-429.1972. 4552999 PMC247423

[ref45] Eilers H.; Pernthaler J.; Glöckner F. O.; Amann R. Culturability and in Situ Abundance of Pelagic Bacteria from the North Sea. Appl. Environ. Microbiol. 2000, 66, 3044–3051. 10.1128/AEM.66.7.3044-3051.2000. 10877804 PMC92109

[ref46] Floyd M. M.; Tang J.; Kane M.; Emerson D. Captured Diversity in a Culture Collection: Case Study of the Geographic and Habitat Distributions of Environmental Isolates Held at the American Type Culture Collection. Appl. Environ. Microbiol. 2005, 71, 2813–2823. 10.1128/AEM.71.6.2813-2823.2005. 15932972 PMC1151842

[ref47] Gärtner A.; Blümel M.; Wiese J.; Imhoff J. F. Isolation and Characterisation of Bacteria from the Eastern Mediterranean Deep Sea. Antonie van Leeuwenhoek 2011, 100, 421–435. 10.1007/s10482-011-9599-5. 21671195

[ref48] Lekunberri I.; Gasol J. M.; Acinas S. G.; Gómez-Consarnau L.; Crespo B. G.; Casamayor E. O.; Massana R.; Pedrós-Alió C.; Pinhassi J. The Phylogenetic and Ecological Context of Cultured and Whole Genome-Sequenced Planktonic Bacteria from the Coastal NW Mediterranean Sea. Syst. Appl. Microbiol. 2014, 37, 216–228. 10.1016/j.syapm.2013.11.005. 24462268

[ref49] Kai W.; Peisheng Y.; Rui M.; Wenwen J.; Zongze S. Diversity of Culturable Bacteria in Deep-Sea Water from the South Atlantic Ocean. Bioengineered 2017, 8, 572–584. 10.1080/21655979.2017.1284711. 28140758 PMC5639861

[ref50] Kearse M.; Moir R.; Wilson A.; Stones-Havas S.; Cheung M.; Sturrock S.; Buxton S.; Cooper S.; Markowitz S.; Duran C.; Thierer T.; Ashton B.; Meinties P.; Drummond A. Geneious Basic: An Integrated and Extendable Desktop Software Platform for the Organization and Analysis of Sequence Data. Bioinformatics 2012, 28, 1647–1649. 10.1093/bioinformatics/bts199. 22543367 PMC3371832

[ref51] Wiegand I.; Hilpert K.; Hancock R. E. W. Agar and Broth Dilution Methods to Determine the Minimal Inhibitory Concentration (MIC) of Antimicrobial Substances. Nat. Protoc. 2008, 3, 163–175. 10.1038/nprot.2007.521. 18274517

[ref52] Zeder M.; Ellrott A.; Amann R. Automated Sample Area Definition for High-Throughput Microscopy. Cytometry, Part A 2011, 79, 306–310. 10.1002/cyto.a.21034. 21412981

[ref53] Zeder M.; Pernthaler J. Multispot Live-Image Autofocusing for High-Throughput Microscopy of Fluorescently Stained Bacteria. Cytometry, Part A 2009, 75, 781–788. 10.1002/cyto.a.20770. 19658173

[ref54] Sprouffske K.; Wagner A. G. An R Package for Obtaining Interpretable Metrics from Microbial Growth Curves. BMC Bioinf. 2016, 17, 172 10.1186/s12859-016-1016-7. PMC4837600 27094401

[ref55] Hall B. G.; Acar H.; Nandipati A.; Barlow M. Growth Rates Made Easy. Mol. Biol. Evol. 2014, 31, 232–238. 10.1093/molbev/mst187. 24170494

[ref56] Altschul S. F.; Gish W.; Miller W.; Myers E. W.; Lipman D. J. Basic Local Alignment Search Tool. J. Mol. Biol. 1990, 215, 403–410. 10.1016/S0022-2836(05)80360-2. 2231712

[ref57] Kozlov A. M.; Darriba D.; Flouri T.; Morel B.; Stamatakis A. RAxML-NG: A Fast, Scalable and User-Friendly Tool for Maximum Likelihood Phylogenetic Inference. Bioinformatics 2019, 35, 4453–4455. 10.1093/bioinformatics/btz305. 31070718 PMC6821337

[ref58] Letunic I.; Bork P. Interactive Tree Of Life (ITOL) v4: Recent Updates and New Developments. Nucleic Acids Res. 2019, 47, W256–W259. 10.1093/nar/gkz239. 30931475 PMC6602468

[ref59] Acinas S. G.; Sánchez P.; Salazar G.; Cornejo-Castillo F. M.; Sebastián M.; Logares R.; Royo-Llonch M.; Paoli L.; Sunagawa S.; Hingamp P.; Ogata H.; Lima-Mendez G.; Roux S.; González J. M.; Arrieta J. M.; Alam I. S.; Kamau A.; Bowler C.; Raes J.; Pesant S.; Bork P.; Agustí S.; Gojobori T.; Vaqué D.; Sullivan M. B.; Pedrós-Alió C.; Massana R.; Duarte C. M.; Gasol J. M. Deep Ocean Metagenomes Provide Insight into the Metabolic Architecture of Bathypelagic Microbial Communities. Commun. Biol. 2021, 4, 604 10.1038/s42003-021-02112-2. 34021239 PMC8139981

[ref60] Duarte C. M. Seafaring in the 21St Century: The Malaspina 2010 Circumnavigation Expedition. Limnol. Oceanogr. Bull. 2015, 24, 11–14. 10.1002/lob.10008.

[ref61] R Core Team A Language and Environment for Statistical Computing; R Foundation for Statistical Computing: Vienna, Austria, 2017, https://www.R-Project.org/.

[ref62] Chiu H.-H.; Shieh W. Y.; Lin S. Y.; Tseng C.-M.; Chiang P.-W.; Wagner-Dö Bler I. Alteromonas Tagae Sp. Nov. and Alteromonas Simiduii Sp. Nov., Mercury-Resistant Bacteria Isolated from a Taiwanese Estuary. Int. J. Syst. Evol. Microbiol. 2007, 57, 1209–1216. 10.1099/ijs.0.64762-0. 17551031

[ref63] Math R. K.; Jin H. M.; Kim J. M.; Hahn Y.; Park W.; Madsen E. L.; Jeon C. O. Comparative Genomics Reveals Adaptation by Alteromonas Sp. SN2 to Marine Tidal-Flat Conditions: Cold Tolerance and Aromatic Hydrocarbon Metabolism. PLoS One 2012, 7, e35784 10.1371/journal.pone.0035784. 22563400 PMC3338528

[ref64] Morishita K.; Nakamura K.; Tuchiya K.; Nishimura K.; Iwahara M.; Yagi O. Removal of Methylmercury from a Fish Broth by Alteromonas Macledii Isolated from Minamata Bay. Jpn. J. Water Treat. Biol. 2006, 42, 45–51. 10.2521/jswtb.42.45.

[ref65] Vetriani C.; Chew Y. S.; Miller S. M.; Yagi J.; Coombs J.; Lutz R. A.; Barkay T. Mercury Adaptation among Bacteria from a Deep-Sea Hydrothermal Vent. Appl. Environ. Microbiol. 2005, 71, 220–226. 10.1128/AEM.71.1.220-226.2005. 15640191 PMC544242

[ref66] De J.; Ramaiah N.; Mesquita A.; Verlekar X. N. Tolerance to Various Toxicants by Marine Bacteria Highly Resistant to Mercury. Mar. Biotechnol. 2003, 5, 185–193. 10.1007/s10126-002-0061-6. 12876655

[ref67] De J.; Ramaiah N. Characterization of Marine Bacteria Highly Resistant to Mercury Exhibiting Multiple Resistances to Toxic Chemicals. Ecol. Indic. 2007, 7, 511–520. 10.1016/J.ECOLIND.2006.05.002.

[ref68] Zheng R.; Wu S.; Ma N.; Sun C. Genetic and Physiological Adaptations of Marine Bacterium Pseudomonas Stutzeri 273 to Mercury Stress. Front. Microbiol. 2018, 9, 682 10.3389/fmicb.2018.00682. 29675016 PMC5895735

[ref69] Robinson J. B.; Tuovinen O. H. Mechanisms of Microbial Resistance and Detoxification of Mercury and Organomercury Compounds: Physiological, Biochemical, and Genetic Analyses. Microbiol. Rev. 1984, 48, 95–124. 10.1128/mr.48.2.95-124.1984. 6377034 PMC373215

[ref70] Lamborg C.; Bowman K.; Hammerschmidt C.; Gilmour C.; Munson K.; Selin N.; Tseng C. M. Mercury in the Anthropocene Ocean. Oceanography 2014, 27, 76–87. 10.5670/oceanog.2014.11.

