# Supplementary material for: Prevalence of Heterotrophic Methylmercury Detoxifying Bacteria across Oceanic Regions
Source: Environ Sci Technol. 2022 Mar 4;56(6):3452–61. doi: 10.1021/acs.est.1c05635 (PMC8928480; doi:10.1021/acs.est.1c05635)
Supplement: Supplementary file 2 — es1c05635_si_002.pdf [file es1c05635_si_002.pdf]

Supplementary Information for

Prevalence of heterotrophic methylmercury detoxifying bacteria across oceanic regions

Isabel Sanz-Sáez, Carla Pereira-García, Andrea G. Bravo, Laura Trujillo, Martí Pla i Ferriol, Miguel Capilla, Pablo Sánchez, Rosa Carmen Rodríguez Martín-Doimeadios, Silvia G. Acinas, Olga Sánchez.

Corresponding authors: Olga Sánchez: olga.sanchez@uab.es; Silvia G. Acinas: sacinas@icm.csic.es

This PDF file includes:

Supplementary Materials and Methods

Supplementary Results

Supplementary Figures S1 to S7

Headings Supplementary Tables S1 to S11

References

Other Supplementary Material for this manuscript include the following:

Excel file with Supplementary Tables S1 to S11.

Supplementary Materials and Methods

Selection of marine strains for merAB functional screening

A total of 2003 marine strains were previously isolated from a wide variety of oceanographic regions and depths. Detailed sampling, isolation procedures and partial sequencing of the 16S rRNA gene have been already described in Sanz-Sáez et al. [1]. This heterotrophic marine bacterial culture collection, called MARINHET, was the basis for the functional screening of Hg resistant bacteria and the description of the distribution of mercury resistance genes of this study. First, we performed a preliminary search of marine genera codifying both *merA* and *merB* in all available finished genomes in the Integrated Microbial Genomes (IMG) database at that time (March 2016) of the Joint Genome Institute (JGI) by: (i) searching the functional annotation of *merA* (as mercuric reductase or mercuric ion reductase) and *merB* (organomercurial lyase or alkylmercury lyase), and (ii) using the Kyoto Encyclopedia of Genes and Genomes (KEGG) orthologs [2] K00520 for *merA* and K00221 for *merB*. Secondly, we downloaded the 16S rRNA gene sequences of those genomes in order to BLASTn [3] them against the partial 16S rRNA sequences of our isolates and to obtain a list of putative candidates carrying the *merA* and *merB* genes. From those, we focused on two genera, *Alteromonas* and *Marinobacter,* highly abundant taxa in our MARINHET cultured collection. A total 290 isolates from both *Alteromonas* (244) and *Marinobacter* (46) genera were finally selected for functional screening of the Hg resistance genes *merA* and *merB*. These strains originated from a variety of oceanographic regions and depths and covered both the photic and aphotic regions of the water column. Information about the origin of the samples where isolates were retrieved is summarized in **Supplementary Table S3**.

Primer design details and requirements

Sequences of the *merA* and *merB* genes were downloaded from Integrated Microbial Genomes (IMG) database of the Joint Genome Institute (JGI) by searching "mercuric reductase" or "mercuric ion reductase" (*merA*) and "organomercurial lyase" or "alkylmercury lyase" (*merB*) genes in the published finished genomes of 22 *Alteromonas* and 6 *Marinobacter* (genomes available in IMG database in 2016). The search resulted in obtaining 15 *merA* and 1 *merB* genes from *Alteromonas* genomes, as well as 9 *merA* and 1 *merB* genes from *Marinobacter* (**Supplementary Table S1**). Specific primer pairs were designed separately for: (i) *merA* of *Alteromonas*, (ii) *merA* of *Marinobacter*, (iii) *merA* + *merB* of *Alteromonas*, and (iv) *merA* + *merB* of *Marinobacter*. Both sets of *merA* primers were selected after alignments of the sequences in Clustal Omega (https://www.ebi.ac.uk/Tools/msa/clustalo/)[4] in order to check for the most conserved areas of the gene. For *Alteromonas*, as different *merA* gene copies could be present

within the same genome, the designed primers only covered the gene/s copies with the highest similarity between them and between the different *Alteromonas* species, since evidence of horizontal gene transfer has been detected in *mer* genes, usually in plasmids, transposons or genomic islands[5] **(Supplementary Table S1)**. On the other hand, as *Marinobacter merA* sequences differ greatly from species to species and most of the *Marinobacter* isolates present in our culture collection taxonomically classified as *Marinobacter hydrocarbonoclasticus, Marinobacter aquaeolei* or *Marinobacter salarius* **(Supplementary Table S2),** primers were designed to cover the *merA* sequence variants of these species (**Supplementary Table S1**). Otherwise, as *merB* genes were only found in *Alteromonas mediterranea* DE strain (CP003917) and *Marinobacter aquaeolei* VT8 strains (NC_008740) (also named *Marinobacter hydrocarbonoclasticus* VT8), *merA* + *merB* sets of primers were designed specifically for these mentioned strains. In this case we used the online tool Primer-BLAST of the National Center for Biotechnology Information (https://www.ncbi.nlm.nih.gov/tools/primer-blast/)[6]. The input sequence for the generation of these primers was a concatenate nucleotide sequence of the *merA* and *merB* genes that were co-localized one next to the other in the *mer* operon (**Supplementary Figure S1A**). All the designed primers had to meet the following requirements: optimum polymerase chain reaction (PCR) product size of 1200 bp for *merA* or 2100 bp for *merA* + *merB* (referred hereafter as *merAB*), annealing temperature around 57 ºC, primers length 20 bp, and 50 % GC content. Designing optimal PCR primer sequences following the above mention parameters allows a successful PCR with sensitive, specific, and reproducible results avoiding cross-hybridization with primers and other gene sequences present in the reaction mixture[7]. In **Supplementary Figure S1B** we summarize the sequences of the different sets of primers used in this study.

PCR details

Each PCR reaction (for *merA* and *merAB* amplification) with a final volume of 25 µl contained: 2 µl of template DNA, 0.5 µl of each deoxynucleotide triphosphate at a concentration of 10 µM, 0.75 µl of $MgCl_2$ 1.5 mM, 0.5 µl of each primer at a concentration of 10 µM, 0.125 µl of Taq DNA polymerase (Invitrogen), 2.5 µl of PCR buffer supplied by the manufacturer (Invitrogen, Paisley, UK) and Milli-Q water up to the final volume. Reactions were carried out in a Biorad thermocycler using the following program: initial denaturation at 94 ºC for 5 min, followed by 30 cycles of 1 min at 94 ºC, 1 min at 55 ºC and 2 min at 72 ºC, and a final extension step of 10 min at 72 ºC.

Phylogenetic trees with aminoacid merA and merAB sequences

On the other hand, phylogenetic trees were also constructed with the amino acid sequences of the amplified *merA* and *merAB* genes. Using Kyoto Encyclopedia of Genes and Genomes (KEGG) identifiers K00520 (for *merA*) and K00221(for *merB*) we retrieved the *merA* and *merB* genes present in UniProtKB. Those sequences were then submitted to BLAST against our detected *merA* and *merB* genes, and best hits were included in the tree as reference sequences. Sequences were aligned with ClustalW of the Geneious software v.11.0.5[8] with the Gonnet substitution matrix and default gap extension and opening penalties as described previously[9]. For *merA*, the dihydrolipoamide dehydrogenase protein sequences from *Magnetospirillum magneticum* AMB-1 (WP_011386317.1) and *Pseudomonas fluorescens* Pf0-1 (WP_011336663.1) served as outgroups. Likewise, we trimmed the N-terminal region of the aligned sequences as in Barkay et al. (2010)[10]. In the case of *merAB*, outgroup sequences were not included. Phylogenetic trees were constructed using maximum-likelihood inference with RAXML-NG 0.9.0[11] and the LG evolutionary model with optimization in the among-site rate heterogeneity model and the proportion of invariant sites (LG+G+I), and 100 bootstrap replicates.

Measurement of the biotic and abiotic degradation of MeHg

In order to characterize the MeHg degradation rates of our bacteria caused by the action of the *merA* and *merB* genes, 2 ml samples were taken from the 1 µM and 5 µM growth curves of the most tolerant strain, *Alteromonas* sp. ISS312, at times 0, 6, 12, 24 and 48 h. Besides, in order to check the possibility that MeHg was being abiotically removed, we measured the MeHg concentrations from samples taken from multiwell plates experiments. These included one well of a liquid culture from the ISS312 strain in Zobell broth (initial O.D. at 600 nm of 0.05) amended with 5 µM CH_3HgCl and incubated at RT during 72 h in the dark, as well as two different controls to detect the possible abiotic degradation of MeHg: (i) medium control (CH_3HgCl at concentrations of 1 µM and 5 µM with no strain added), and (ii) killed control (CH_3HgCl at concentrations of 1 µM and 5 µM added to autoclaved liquid cultures of the strain). Three replicates of 2 ml samples from each well, containing the CH_3HgCl treatment and the different controls, were taken at times 0 and 72 h. Collected samples were immediately frozen at -80 ºC. Concentration of Hg species was measured by direct derivatization of the culture samples with sodium tetraethyl borate and injection into a hyphenated system consisting of a gas chromatograph coupled to an atomic fluorescence detector via pyrolysis (GC-pyro-AFS) as previously described elsewhere [12]. Briefly, 2 ml samples were used for derivatization. The pH of the extracts was adjusted to 3.9 by adding 5 ml of 0.1 M acetic acid-sodium acetate buffer and ammonia (20 %) if necessary. Then, 2 ml of hexane and 250 µl of sodium tetraethyl borate (6 %,

w/v) were added and the mixture was manually shaken for 5 min. The sample was centrifuged for 5 min at 600 g. The organic layer was transferred to a chromatographic glass vial and stored at -18 ºC until analysis. When Hg species were not detectable, the organic layer was preconcentrated under a gentle stream of nitrogen to a low volume (50-100 µL) just before the measurement. The procedural detection limits, after pre-concentration, were 0.19 and 0.23 nM for MeHg and inorganic Hg, respectively.

Strain ISS312 genome sequencing

DNA from strain ISS312 was extracted using the DNeasy Blood & Tissue kit (Qiagen), following the manufacturer's recommendations. A genome library was prepared with a Celero™ DNA-seq library system and sequencing was performed with paired-ended 300 bp long reads by IGA-Tech with a MiSeq Illumina machine. The sequence data was filtered to remove the adapters and the unpaired reads with *cutadapt* v.1.16 [13] and the quality was assessed before and after with *fastaqc* v.0.11.7. The clean data were used to do the assembly with Spades v.3.12.0 and then optimized with the same program. QUAST v.5.0.1 [14] and ALE were used to assess the quality of the assemblies and the best scores were selected. K-mer 121, 125 and 127 were selected for the optimized-combined assembly and the quality was assessed again. The annotation was done with Prokka v1.13 [15] and the completeness of the genome was checked with CheckM v1.0.18 [16]. In order to search for plasmids within our contigs, we used the database PLSDB [17].

Fragment recruitment analysis of the genome of strain ISS312 and MAG-0289 in bathypelagic metagenomes

The abundance of ISS312 across the global bathypelagic ocean was assessed thanks to its complete genome. Fragment Recruitment Analysis (FRA) was performed by mapping the metagenomic reads of 58 bathypelagic microbial metagenomes from 32 stations [18] from Malaspina Expedition, including free-living (0.2-0.8 µm) and particle-attached (0.8-20 µm) bacterial communities. Analyses were done with BLASTn v2.7.1+ [3] using the following alignment parameters*: -perc_identity* 70, *-evalue* 0.0001. Only those reads with more than 90 % coverage and mapping identity equal to or higher than 95 % were kept for analysis. In order to remove possible false mapping hits to the conserved regions of rRNA genes, reads aligning to the regions annotated as ribosomal genes were not considered for the analysis. Read counts from mapped reads from each metagenome were corrected by their sequencing depth to make them comparable through samples.

The abundance of the MAG-0289 across the Malaspina bathypelagic dataset was done as described above, but with a previous subsampling step to the shallower sequencing depth (4,175,346 read pairs) with *bbtools reformat*.sh v.38.08 (https://sourceforge.net/projects/bbmap/). Here, when reads could map with the same probability to any of the genomes (same *e-value*, same alignment length and identity), they were assigned at random. The average nucleotide identity (ANI) of ISS312 against MAG-0289 was calculated with fastANI v1.2 as both were related to the *Alteromonas mediterranea* species.

Preparation and observation of samples for transmission electron microscopy

In order to study potential effects of MeHg on strain ISS312 cells morphology, the isolate was grown separately during 24 h in Zobell broth not amended with CH_3HgCl and amended at a final concentration of 5 µM with shaking in the dark. The overnight culture was centrifuged at 1000 g during 15 min and the supernatant was discarded. The pellet was fixed with paraformaldehyde 2 % final concentration during 30 min at RT. After fixation, the pellet was processed as previously described [19] to finally obtain thin sections of the samples that were examined by transmission electron microscopy (TEM, JEM-1400 plus, JEOL). Visualizations were done by the microscopy service of the *Universitat Autònoma de Barcelona* (http://sct.uab.cat/microscopia/en/content/inici).

Supplementary Results

Exploring the MARINHET culture collection

We detected a total of 20 different bacterial taxa in the IMG/JGI database that matched at the genera level with the taxonomic assignation of the MARINHET culture collection isolates, and therefore, with potential for carrying *merA* and *merB* genes (**Supplementary Table S9**). The comparative analyses between the 16S rRNA gene sequences of these 20 genera containing the targeted *merA* and *merB* genes and the partial 16S rRNA sequences of our isolates revealed a total of 352 strains that were, at least, 99 % identical to one of the putative candidates genera. These comprised 7 genera (*Alteromonas, Marinobacter, Idiomarina, Pseudomonas, Micrococcus, Zunongwangia and Bacillus*) (**Supplementary Table S10**). From these, we selected a total of 244 strains affiliating to *Alteromonas sp.* and 46 strains to *Marinobacter sp.* (**Supplementary Table S2**) for performing the functional screening of *merA* and *merB* genes through PCR.

Ribosomal phylogenetic reconstruction of strains codifying merA and/or merB genes

The phylogenetic analyses of the 16S rRNA gene from all of the detected strains harboring *merA* and *merAB* genes, together with the rest of the screened strains, revealed some patterns (**Supplementary Figure S7**). First, most of the isolated *Alteromonas* strains with *merA* were related to *Alteromonas australica* and *Alteromonas mediterranea*, and only strains affiliating to the last one presented both genes *merA* and *merB (merAB)*. We also detected one strain with *merAB* genes affiliated to *Alteromonas macleodii.* Secondly, *Marinobacter* strains displaying *merAB* were related to *Marinobacter hydrocarbonoclasticus, Marinobacter salarius* and some uncultured *Marinobacter* strains. We are aware that our primers do not match the whole diversity within *Alteromonas* since there is a large uncultured *Alteromonas* cluster related to NW Mediterranean, Indian Ocean and North and South Atlantic samples and from photic and aphotic layers that was not covered by our primer-set (**Supplementary Figure S7**). Moreover, this phylogeny showed that positive strains for *merA* and *merB* genes clustered together with strains which do not harbor these genes (**Supplementary Figure S7**). It has been described that some bacterial species codify different sequence variants of the *merA* gene [9,20] including the *Alteromonas* genus [5]. Therefore, it is possible that some of the *Alteromonas* and *Marinobacter* strains within the MARINHET collection tested presented different sequence variants, although we were only able to detect the ones targeted by the primers designed. On the other hand, the operon *mer* can be either codified in the chromosome [21] or in plasmids [22,23], and usually, *mer* genes are components of transposons [24], and integrons [25,26]. Thus, it is not surprising to find some strains within the same species without the *mer* operon.

Results from merA and merAB phylogenies

The amino acid *merA* phylogeny, which theoretically could include the different gene sequence variants covered by the primers designed, unveiled that all the *Alteromonas merA* sequences grouped into two sister clades (tree branches colored in blue) with the reference *Alteromonas mediterranea merA* sequence **(Supplementary Figure S2).** MIC variability was more clearly observed here as sequences clustering together presented MIC values ranging from 10 µM to 70 µM. For *Marinobacter,* the *merA* genes grouped into three different clusters (tree branches colored distinctively) showing genetic heterogeneity among their *merA* genes copies (**Supplementary Figure S2**). In the case of the *merAB* aminoacids phylogeny, sequences clustered all together, as expected, with *Alteromonas mediterranea* or *Marinobacter hydrocarbonoclasticus* VT8 reference sequences **(Supplementary Figure S3)**.

Supplementary Figures

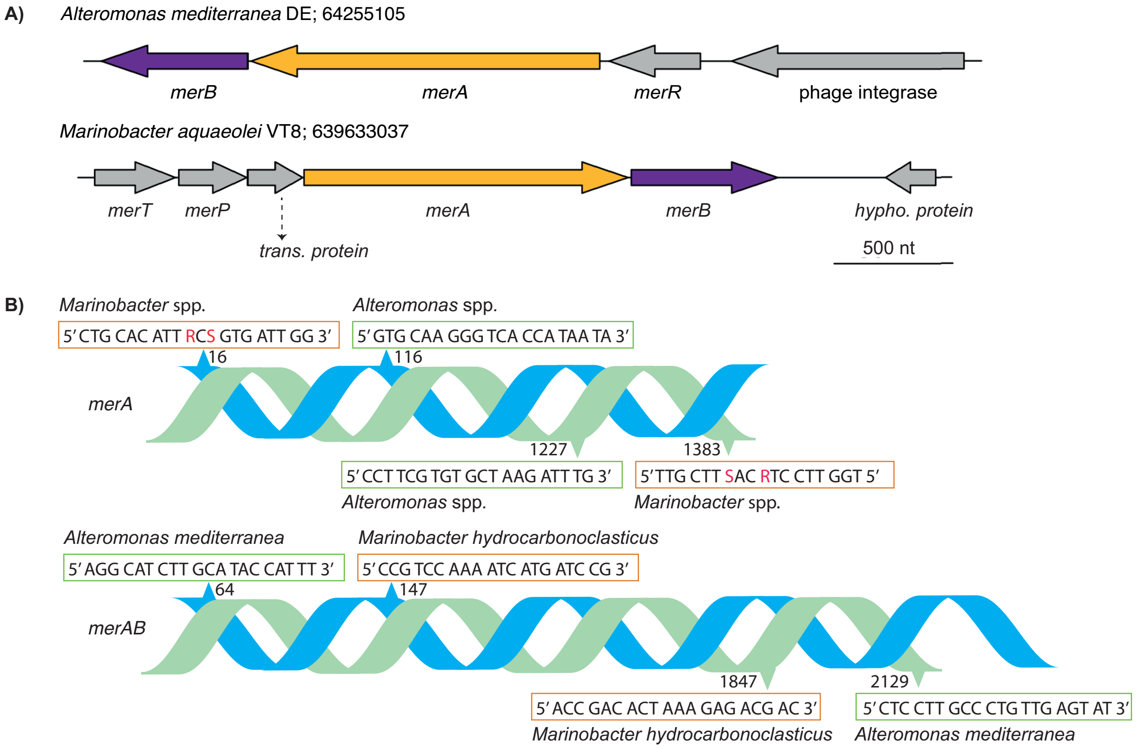

Figure S1. Synteny and primers of *merA* and *merB* genes. (A) Co-localization of *merA* and *merB* genes in *Alteromonas mediterranea* DE and *Marinobacter aquaeolei* VT8. Coordinates extracted from the JGI/IMG database. Accession number in the IMG/JGI database is indicated with the species name. Genes: *merA*, mercuric reductase; *merB*, organomercurial lyase; *merR*, *mer* operon regulator; *merT*, *merP* and trans. prot.: mercury transport proteins; hypho. prot: hypothetical protein. **(B)** Forward (blue chain) and reverse (green chain) primers sequences for *merA* and *merAB* genes. Numbers indicate the position where primers attached to the forward and reverse DNA strands. Some of the primers display degenerated bases (see in red): R: A or G; S: G or C.

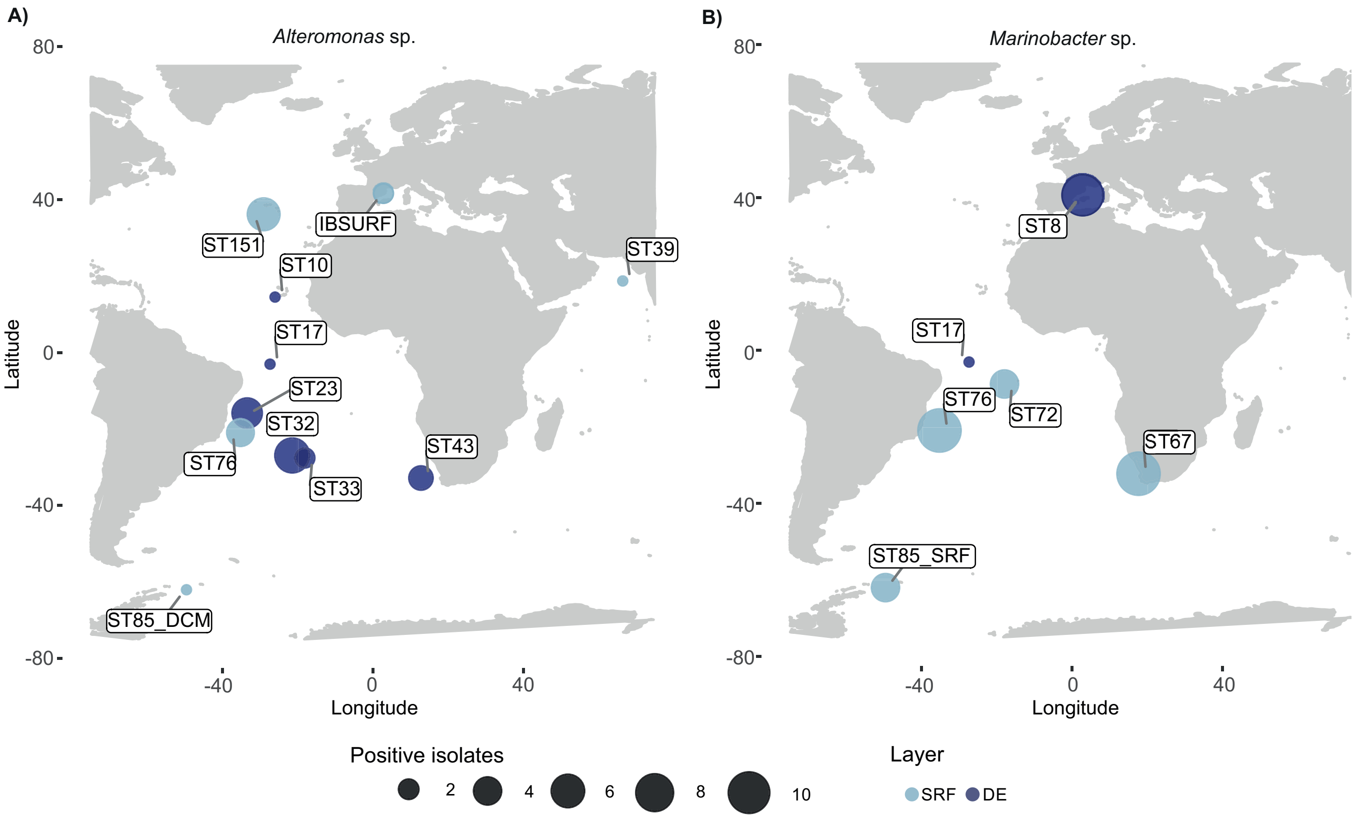

Figure S2. Distribution of the positive strains after functional screening for *merA* and/or *merAB* genes. (A) *Alteromonas* sp. strains **(B)** *Marinobacter* sp. strains. Size of the dots indicates how many strains per station including *merA* and/or *merAB* were identified. Color of the dots indicated the layer (photic or bathypelagic) where the strains were retrieved. SRF: surface, DCM; deep-chlorophyll maximum; DE: bathypelagic.

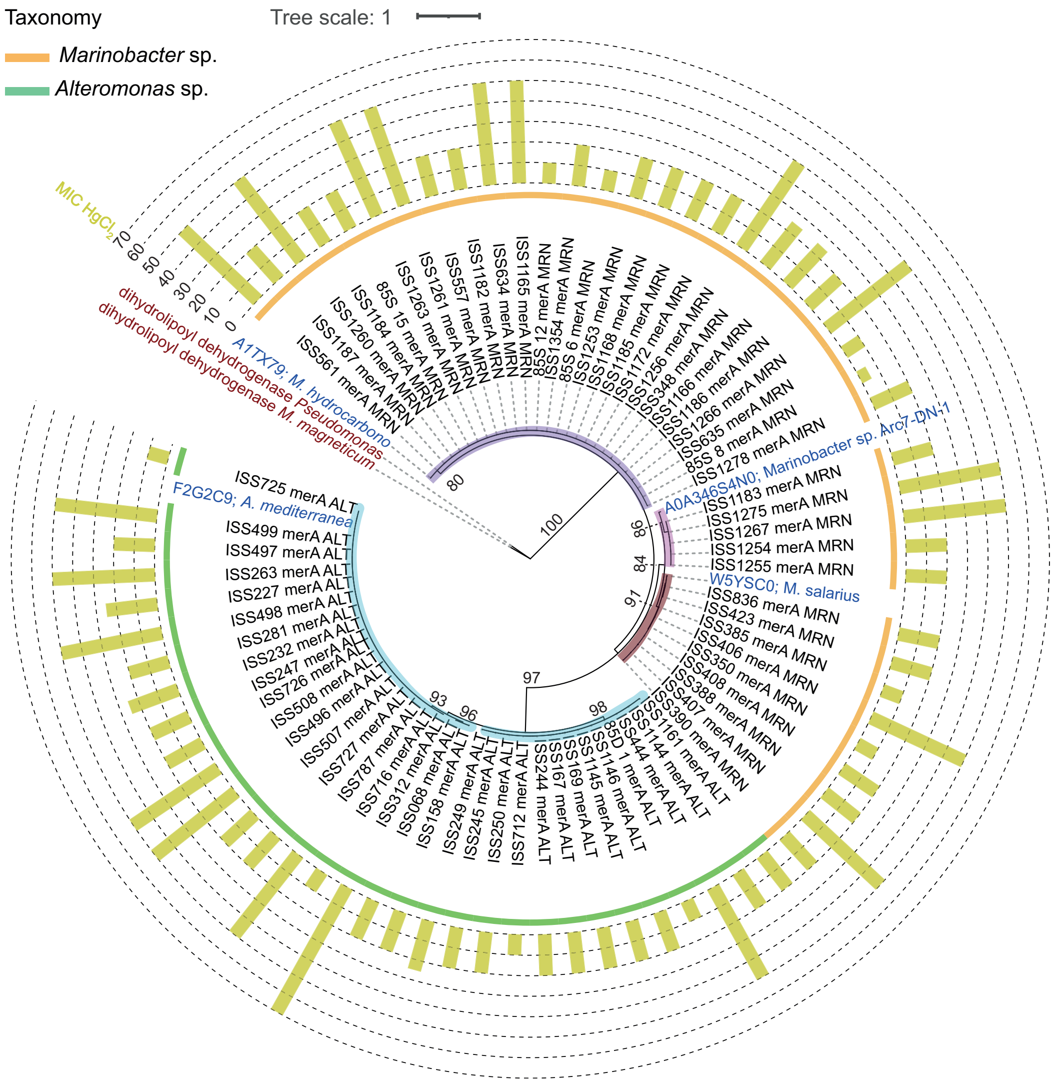

Figure S3. Phylogeny of the *merA* amino acid sequences from *Alteromonas* and *Marinobacter* positive strains. Including only those sequences codified in those strains submitted to Minimum Inhibitory Concentration (MIC) assays. MIC results for the tested strains against $HgCl_2$ are indicated by bars. Sequences of dihydrolipoyl dehydrogenase act as outgroups of the tree, M. *magneticum* as *Magnetospirillum magneticum*. Color strip indicates taxonomy of the sequences**:** *Alteromonas* sp., green; *Marinobacter* sp., orange. Reference sequences are indicated in bold blue: *A. mediterranea, Alteromonas mediterranea; M. hydrocarbonoclasticus., Marinobacter hydrocarbonoclasticus; M. salarius, Marinobacter salarius.* The numbers in the nodes represent bootstrap percentages > 75%. Identical colored branches represent sister clades while branches colored distinctively indicate different clades.

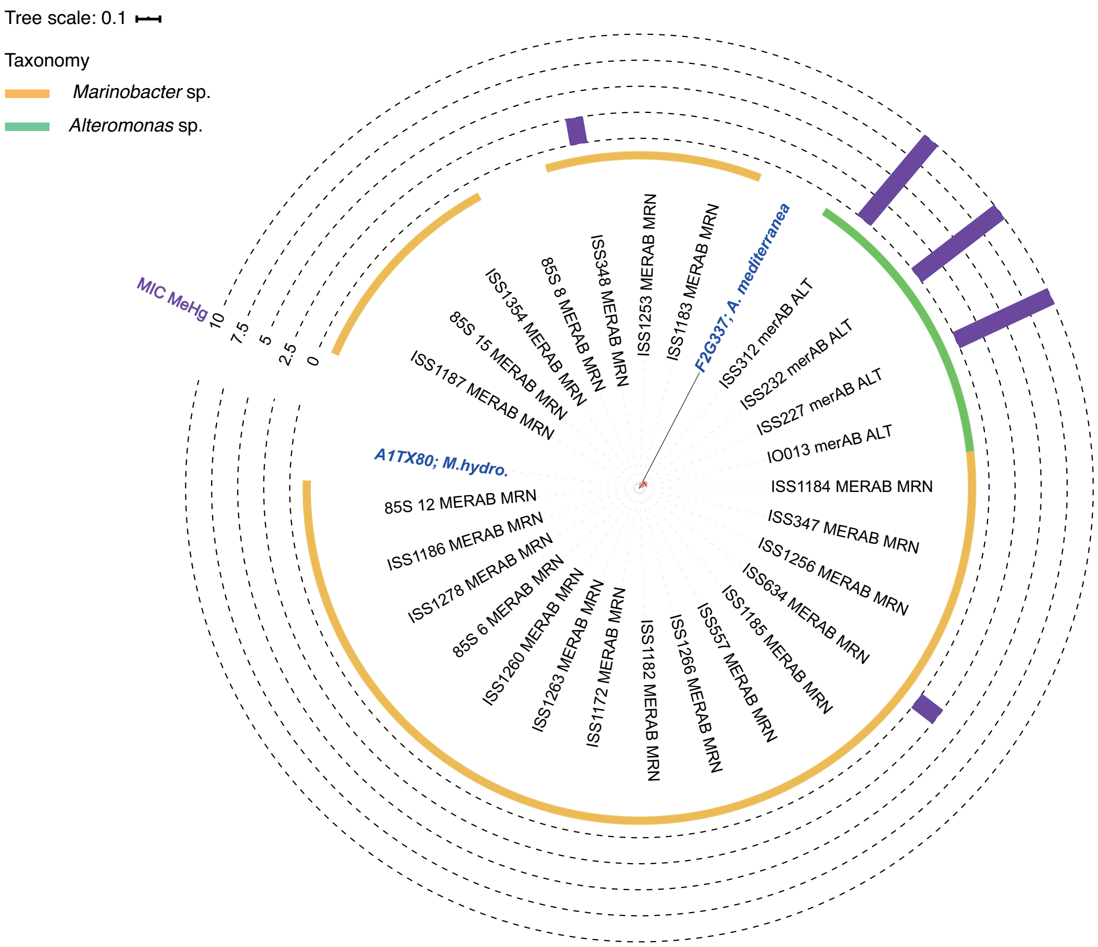

Figure S4. Phylogenetic tree inferred with the *merAB* amino acid sequences. Color strip indicates taxonomy of the sequences**:** *Alteromonas* sp., green; *Marinobacter* sp., orange. Reference sequences are indicated in bold blue: *A. mediterranea, Alteromonas mediterranea; M. hydro., Marinobacter hydrocarbonoclasticus.* MIC results for the tested strains against MeHg are indicated by bars. Bootstrap values >75% are indicated by red circles in the tree nodes.

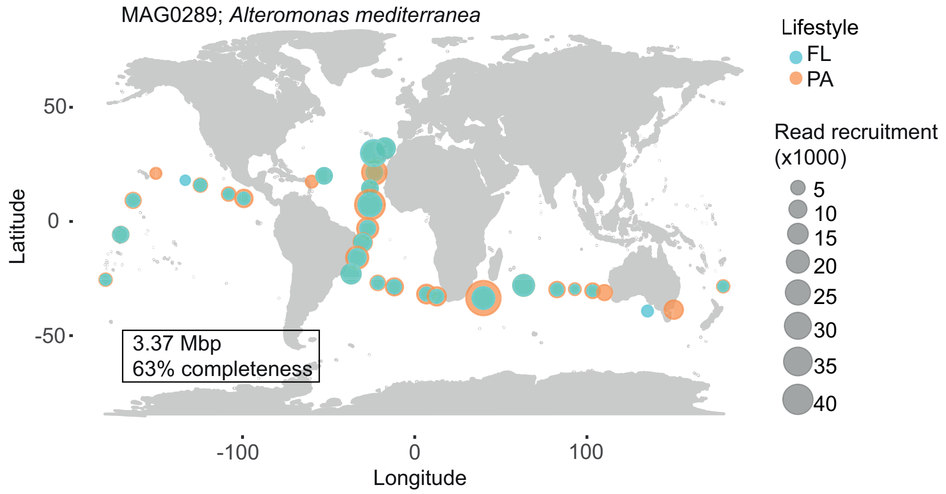

Figure S5. World map showing the distribution of MAG-0289 in bathypelagic marine metagenomes. Size of the dots indicate number of reads (x1000) and color indicate if the reads recruited in the free-living (FL) or in the particle-attached (PA) bacterial communities of the bathypelagic samples.

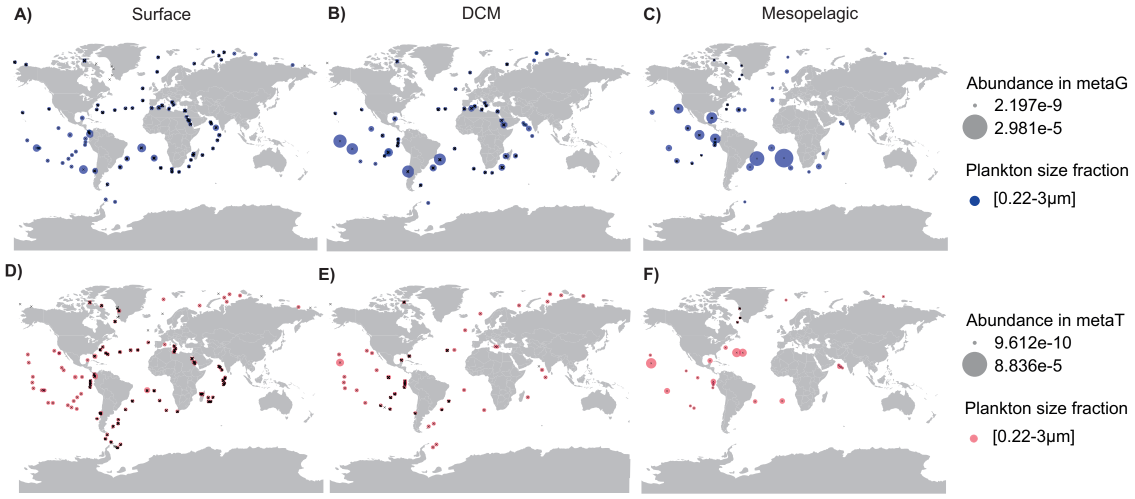

Figure S6. Biogeography of *merA* genes across oceanic regions from microbial metagenomes (metaG) and metatranscriptomes (metaT) from *Tara* Oceans Expedition. (A,B,C). Abundance of *merA* genes found in the Surface, DCM and Mesopelagic metagenomes. **(D,E,F).** Abundance of *merA* transcripts found in the Surface, DCM and Mesopelagic metatranscriptomes.

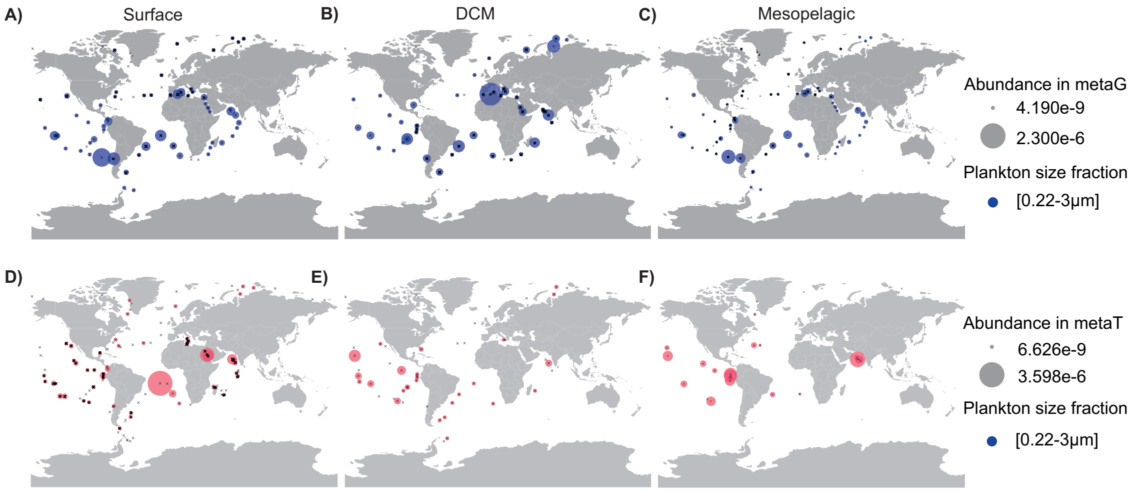

Figure S7. Biogeography of *merB* genes across oceanic regions from microbial metagenomes (metaG) and metatranscriptomes (metaT) from *Tara* Oceans Expedition. (A,B,C). Abundance of *merB* genes found in the Surface, DCM and Mesopelagic metagenomic samples. **(D,E,F).** Abundance of *merB* transcripts found in the Surface, DCM and Mesopelagic metatranscriptomics samples.

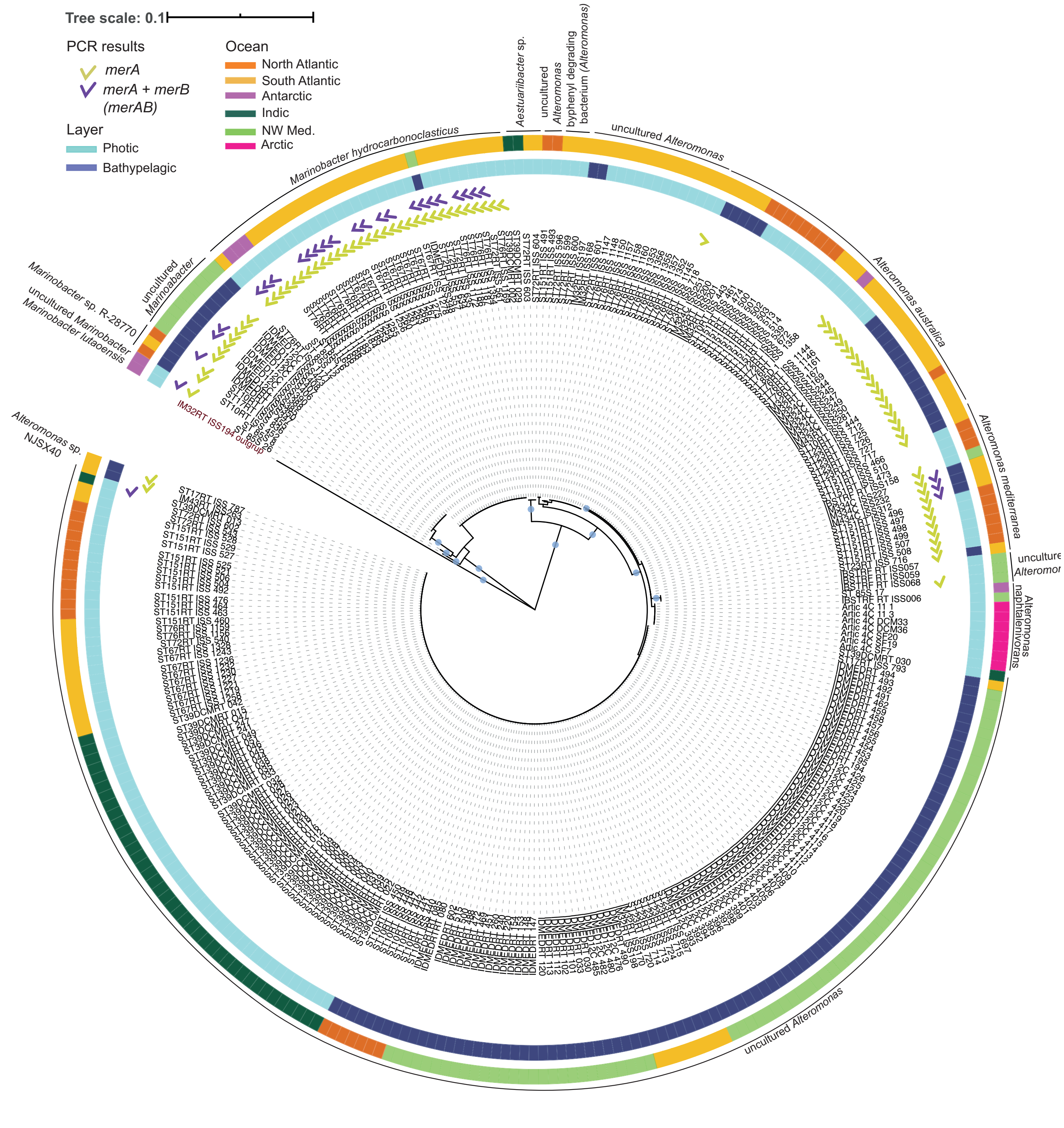

Figure S8. Phylogenetic tree with the 16S rRNA gene sequences of all the isolates PCR screened for *merA* and *merAB* genes. Presence or absence of both *merA* and *merAB* genes is indicated by tick symbols. Depth (first color strip) and oceanographic location (second color strip) where the isolates were retrieved are indicated by colors. Bootstrap values ≥ 75 are indicated by blue circles in the tree nodes. For graphic representation reference 16S rRNA sequences were removed from original tree, and names of these references sequences have been placed surrounding tree labels.

Headings Supplementary Tables

Table S1. Downloaded *merA* and *merB* sequences from the JGI/IMG database (2016) to design the PCR primers. In green are selected those *merA* and *merB* copies that should be recognized by the primers designed.

Table S2. Information of the *Alteromonas sp.* and *Marinobacter* sp. strains used for PCR screening of the *merA* and *merB* genes.

Table S3. Characteristics of the marine seawater samples from five different cruises used for the isolation of bacteria screened for Hg resistance genes and results from PCR screening. ATP09: Arctic Tipping Points cruise in 2009; BBMO: Blanes Bay Microbial Observatory.

Table S4. Summary of the number of positive strains per oceanographic region.

Table S5. Summary of the results for the MIC determination assays to $HgCl_2$.

Table S6. Concentrations of MeHg and inorganic mercury (IHg) at different time points during the growth curves at 1 μM and 5 μM. SD: standard deviation; LOD: limit of detection.

Table S7. Biotic (culture) and abiotic controls (killed and medium alone) of the 5 μM MeHg degradation performed by the ISS312 *Alteromonas* strain. SD: standard deviation; LOD: limit of detection.

Table S8. Taxonomic classification of the 54 *merA* and 6 *merB* gene homologous to *merA* and *merB* genes from ISS312 strain found in global metagenomes and metatranscriptomes from *Tara* Oceans expedition.

Table S9. Candidate genera for mercury bioremediation obtained from the search of *merA* and *merB* genes in the KEGG database and from the IMG/JGI database.

Table S10. Summary of the putative isolates within the MARINHET culture collection that could harbor *merA* and *merB* genes based on the BLASTn search between the partial 16S rRNA genes sequences of the isolates and the 16S rRNA gene sequences of the candidate genera extracted from the JGI/IMG database.

References

(1) Sanz-Sáez, I.; Salazar, G.; Sánchez, P.; Lara, E.; Royo-Llonch, M.; Sà, E. L.; Lucena, T.; Pujalte, M. J.; Vaqué, D.; Duarte, C. M.; Gasol, J. M.; Pedrós-Alió, C.; Sánchez, O.; Acinas, S. G. Diversity and Distribution of Marine Heterotrophic Bacteria from a Large Culture Collection. *BMC Microbiol.* **2020**, *20* (1), 207. https://doi.org/10.1186/s12866-020-01884-7.

(2) Kanehisa, M.; Goto, S. KEGG: Kyoto Encyclopedia of Genes and Genomes. *Nucleic Acids Res.* **2000**, *28* (1), 27–30. https://doi.org/10.1093/nar/28.1.27.

(3) Altschul, S. F.; Gish, W.; Miller, W.; Myers, E. W.; Lipman, D. J. Basic Local Alignment Search Tool. *J. Mol. Biol.* **1990**, *215* (3), 403–410. https://doi.org/10.1016/S0022-2836(05)80360-2.

(4) Madeira, F.; Park, Y. M.; Lee, J.; Buso, N.; Gur, T.; Madhusoodanan, N.; Basutkar, P.; Tivey, A. R. N.; Potter, S. C.; Finn, R. D.; Lopez, R. The EMBL-EBI Search and Sequence Analysis Tools APIs in 2019. *Nucleic Acids Res.* **2019**, *47* (W1), W636–W641. https://doi.org/10.1093/nar/gkz268.

(5) Ivars-Martinez, E.; Martin-Cuadrado, A.-B.; D'Auria, G.; Mira, A.; Ferriera, S.;

Johnson, J.; Friedman, R.; Rodriguez-Valera, F. Comparative Genomics of Two Ecotypes of the Marine Planktonic Copiotroph Alteromonas Macleodii Suggests Alternative Lifestyles Associated with Different Kinds of Particulate Organic Matter. *ISME J.* **2008**, *2* (12), 1194–1212. https://doi.org/10.1038/ismej.2008.74.

(6) Ye, J.; Coulouris, G.; Zaretskaya, I.; Cutcutache, I.; Rozen, S.; Madden, T. L. Primer-BLAST: A Tool to Design Target-Specific Primer for Polymerase Chain Reaction. *BMC Bioinformatics* **2012**, *13* (134).

(7) Mitsuhashi, M. Technical Report: Part 1. Basic Requirements for Designing Optimal Oligonucleotide Probe Sequences. *J. Clin. Lab. Anal.* **1996**, *10* (5), 277–284. https://doi.org/10.1002/(SICI)1098-2825(1996)10:5<277::AID-JCLA8>3.0.CO;2-5.

(8) Kearse, M.; Moir, R.; Wilson, A.; Stones-Havas, S.; Cheung, M.; Sturrock, S.; Buxton, S.; Cooper, S.; Markowitz, S.; Duran, C.; Thierer, T.; Ashton, B.; Meinties, P.; Drummond, A. Geneious Basic: An Integrated and Extendable Desktop Software Platform for the Organization and Analysis of Sequence Data. *Bioinformatics* **2012**, *28* (12), 1647–1649.

(9) Boyd, E. S.; Barkay, T. The Mercury Resistance Operon: From an Origin in a Geothermal Environment to an Efficient Detoxification Machine. *Front. Microbiol.* **2012**, *3* (October), 349. https://doi.org/10.3389/fmicb.2012.00349.

(10) Barkay, T.; Kritee, K.; Boyd, E.; Geesey, G. A Thermophilic Bacterial Origin and Subsequent Constraints by Redox, Light and Salinity on the Evolution of the Microbial Mercuric Reductase. *Environ. Microbiol.* **2010**, *12* (11), 2904–2917. https://doi.org/10.1111/j.1462-2920.2010.02260.x.

(11) Kozlov, A. M.; Darriba, D.; Flouri, T.; Morel, B.; Stamatakis, A. RAxML-NG: A Fast, Scalable and User-Friendly Tool for Maximum Likelihood Phylogenetic Inference. *Bioinformatics* **2019**, *35* (21), 4453–4455. https://doi.org/10.1093/bioinformatics/btz305.

(12) Berzas Nevado, J. J.; Rodríguez Martín-Doimeadios, R. C.; Krupp, E. M.; Guzmán Bernardo, F. J.; Rodríguez Fariñas, N.; Jiménez Moreno, M.; Wallace, D.; Patiño Ropero, M. J. Comparison of Gas Chromatographic Hyphenated Techniques for Mercury Speciation Analysis. *J. Chromatogr. A* **2011**, *1218* (28), 4545–4551. https://doi.org/10.1016/J.CHROMA.2011.05.036.

(13) Martin, M. Cutadapt Removes Adapter Sequences from High-Throughput Sequencing Reads. *EMBnet.journal* **2021**, *17* (1), 10–12.

(14) Gurevich, A.; Saveliev, V.; Vyahhi, N.; Tesler, G. QUAST: Quality Assessment Tool for Genome Assemblies. *Bioinformatics* **2013**, *29* (8), 1072–1075. https://doi.org/10.1093/bioinformatics/btt086.

(15) Seemann, T. Prokka: Rapid Prokaryotic Genome Annotation. *Bioinformatics* **2014**, *30* (14), 2068–2069. https://doi.org/10.1093/bioinformatics/btu153.

(16) Parks, D. H.; Imelfort, M.; Skennerton, C. T.; Hugenholtz, P.; Tyson, G. W. CheckM: Assessing the Quality of Microbial Genomes Recovered from Isolates, Single Cells, and Metagenomes. *Genome Res.* **2015**, *25* (7), 1043–1055. https://doi.org/10.1101/gr.186072.114.

(17) Galata, V.; Fehlmann, T.; Backes, C.; Keller, A. PLSDB: A Resource of Complete Bacterial Plasmids. *Nucleic Acids Res.* **2019**, *47* (D1), D195–D202. https://doi.org/10.1093/nar/gky1050.

(18) Duarte, C. M. Seafaring in the 21St Century: The Malaspina 2010 Circumnavigation Expedition. *Limnol. Oceanogr. Bull.* **2015**, *24* (1), 11–14. https://doi.org/10.1002/lob.10008.

(19) Lee, C.; Kim, J. Y.; Lee, W. Il; Nelson, K. L.; Yoon, J.; Sedlak, D. L. Bactericidal Effect of Zero-Valent Iron Nanoparticles on Escherichia Coli. *Environ. Sci. Technol.* **2008**, *42* (13), 4927–4933. https://doi.org/10.1021/es800408u.

(20) Harada, M.; Ito, K.; Nakajima, N.; Yamamura, S.; Tomita, M.; Suzuki, H.; Amachi, S. Genomic Analysis of Pseudomonas Sp. Strain SCT, an Iodate-Reducing Bacterium Isolated from Marine Sediment, Reveals a Possible Use for Bioremediation. *G3* **2019**, *9* (5), 1321–1329. https://doi.org/10.1534/g3.118.200978.

(21) Math, R. K.; Jin, H. M.; Kim, J. M.; Hahn, Y.; Park, W.; Madsen, E. L.; Jeon, C. O.

Comparative Genomics Reveals Adaptation by Alteromonas Sp. SN2 to Marine Tidal-Flat Conditions: Cold Tolerance and Aromatic Hydrocarbon Metabolism. *PLoS One* **2012**, *7* (4), e35784. https://doi.org/10.1371/journal.pone.0035784.
(22) Griffin, H. G.; Foster, T. J.; Silver, S.; Misra, T. K. Cloning and DNA Sequence of the Mercuric- and Organomercurial-Resistance Determinants of Plasmid PDU1358. *Proc. Natl. Acad. Sci. U. S. A.* **1987**, *84* (10), 3112–3116. https://doi.org/10.1073/pnas.84.10.3112.
(23) Barkay, T.; Miller, S. M.; Summers, A. O. Bacterial Mercury Resistance from Atoms to Ecosystems. *FEMS Microbiol. Rev.* **2003**, *27* (2–3), 355–384. https://doi.org/10.1016/S0168-6445(03)00046-9.
(24) Mindlin, S.; Kholodii, G.; Gorlenko, Z.; Minakhina, S.; Minakhin, L.; Kalyaeva, E.; Kopteva, A.; Petrova, M.; Yurieva, O.; Nikiforov, V. Mercury Resistance Transposons of Gram-Negative Environmental Bacteria and Their Classification. *Res. Microbiol.* **2001**, *152* (9), 811–822. https://doi.org/10.1016/S0923-2508(01)01265-7.
(25) Osborn, A. M.; Bruce, K. D.; Strike, P.; Ritchie, D. A. Distribution, Diversity and Evolution of the Bacterial Mercury Resistance (Mer) Operon. *FEMS Microbiol. Rev.* **1997**, *19* (4), 239–262. https://doi.org/10.1016/S0168-6445(97)00003-X.
(26) Bass, L.; Liebert, C. A.; Lee, M. D.; Summers, A. O.; White, D. G.; Thayer, S. G.; Maurer, J. J. Incidence and Characterization of Integrons, Genetic Elements Mediating Multiple-Drug Resistance, in Avian Escherichia Coli. *Antimicrob. Agents Chemother.* **1999**, *43* (12), 2925–2929.
